# Network Flow Methods for NMR-Based Compound Identification

**DOI:** 10.1021/acs.analchem.4c01652

**Published:** 2025-02-25

**Authors:** Leonhard Lücken, Nico Mitschke, Thorsten Dittmar, Bernd Blasius

**Affiliations:** † Institute for Chemistry and Biology of the Marine Environment (ICBM), Carl von Ossietzky Universität Oldenburg, Ammerländer Heerstraße 114-118, 26129 Oldenburg, Germany.; ‡ Helmholtz Institute for Functional Marine Biodiversity, Carl von Ossietzky Universität Oldenburg, Ammerländer Heerstraße 114-118, 26129 Oldenburg, Germany

## Abstract

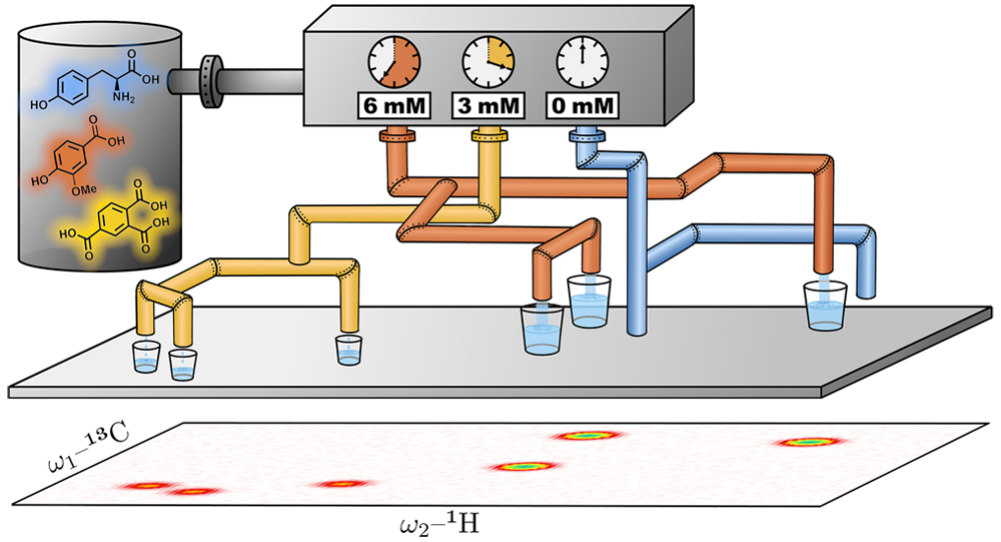

In this work, we introduce a novel method for compound identification in mixtures based on nuclear magnetic resonance spectra. Contrary to many other methods, our approach can be used without peak-picking the mixture spectrum and simultaneously optimizes the fit of all individual compound spectra in a given library. At the core of the method, a minimum cost flow problem is solved on a network consisting of nodes that represent spectral peaks of the library compounds and the mixture. We show that our approach can outperform other popular algorithms by applying it to a standard compound identification task for 2D ^1^H,^13^C HSQC spectra of artificial mixtures and a natural sample using a library of 501 compounds. Moreover, our method retrieves individual compound concentrations with at least semiquantitative accuracy for artificial mixtures with up to 34 compounds. A software implementation of the minimum cost flow method is available on GitHub (https://github.com/GeoMetabolomics-ICBM/mcfNMR).

## Introduction

Liquid-state nuclear magnetic resonance (NMR) spectroscopy is a well-established and powerful technique to elucidate molecular structures. In recent years, increasing effort has been directed to the application of NMR spectroscopy for compound identification and quantification.^1,2^ Methods in this context analyze the most important features of an NMR spectrum, which are the positions and total intensities of resonance peaks, referred to as chemical shifts and integrals, respectively. For more technical information on NMR spectroscopy we refer to one of the numerous publications^3,4^ or classical textbooks^5,6^ on this topic.

Although affected by the measurement matrix and conditions, in first approximation, the NMR spectrum of a mixture can be considered as the sum of the individual spectra of its constituents. In principle, the problem of identifying all contained compounds is therefore solvable by finding the linear recombination of individual compound spectra that best approximates the mixture’s spectrum. Nevertheless, several factors can impede a complete reconstruction. First of all, the mixture may contain compounds whose spectral peak pattern is not known. This is a problem of empirical knowledge. A more fundamental difficulty is posed by overlapping peaks. Since peaks have a considerable extension, such overlaps occur frequently in complex mixtures. Although spectral deconvolution methods can significantly improve the distinction of overlapping peaks,^7^ their potential is limited.^8^ Many peak overlaps in one-dimensional (1D) spectra can be resolved by recording two-dimensional (2D) or even three-dimensional (3D) NMR spectra. However, their acquisition times are usually considerably longer and, for more complex samples, peak overlapping also occurs in 2D and 3D spectra.

Another difficulty is that peaks may shift, change their shape or even disappear, depending on the sample matrix and temperature.^9−11^ This requires an appropriate reconstruction method that is tolerant toward peak disturbance. However, tolerance increases the probability of false identification. The aim to lower this probability while capturing as many contained compounds as possible has driven recent efforts to automate this process.

All available computational tools for NMR-based compound identification operate with a library of individual compound spectra, which are either experimentally measured or mathematically predicted. After the user provides a target spectrum (also referred to as “query spectrum”), one or several candidate spectra from the library are returned based on the comparison of their peaks with mixture peaks.^12−14^ To our knowledge, all existing methods for compound identification in 2D spectra rely on peak picking as a preprocessing step for the spectral data. This step converts the matrix of intensity values on a grid over the spectral domain (we will refer to this as “grid data”) into a list containing information about discrete peaks, such as location and integral. Although peak picking is usually assisted by software, the results are frequently reviewed manually.

In this work, we introduce a novel family of methods for the NMR-based reconstruction of complex mixtures, which can be applied to both grid data and peak lists of arbitrary dimensionality. Our approach is inspired by the Earth Mover’s Distance (EMD), which is also known as Kantorovich or Wasserstein distance.^15^ In the context of NMR analysis, Zhang et al.^16^ have employed it for assessing the similarity of experimental and predicted spectra for individual compounds. Promising EMD-based approaches have been developed for estimating proportions of compounds in a mixture by minimizing the EMD of the normalized mixture spectrum and the superposition of the individual compound spectra.^17−19^ Being based on an efficient calculation of the EMD in one dimension, these approaches can only be used for 1D spectra. In this work, we adapt and extend the underlying methodology for the task of mixture reconstruction using spectra of higher dimensionality and demonstrate its feasibility for the analysis of more complex mixtures. By finding a minimum cost flow (MCF)^20^ on a network tailored to the problem, our approach fits all library spectra simultaneously to the target spectrum. This allows accounting for dependencies between compounds, which may remain ignored if compounds are fitted separately. Since the resulting compound assignment flows are fully quantitative, MCF methods bear the potential to quantitatively reconstruct a mixture spectrum. Given these features, network flow techniques offer a promising new approach to the problem of compound identification and quantification in NMR data.

Following a more detailed introduction of the MCF method given in the next section, we compare its performance on a standard classification task, mapping a library of 501 compounds onto artificial mixtures of 21–27 compounds,^12^ with other popular methods (MetaboMiner,^12^ COLMAR-HSQC,^13^ and SMART-Miner^14^). Further, we test these methods on a plasma sample, and evaluate the ability of MCF methods to quantitatively reconstruct compound concentrations using a library of 34 compounds for a test set of mixture spectra.

## Methods

### Mixture Reconstruction

Whether represented by grid data or by a peak list, an NMR spectrum can formally be written as
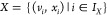
1where we use indices from an index set *I*_*X*_ to unambiguously refer to peaks of *X*, with *x*_*i*_ and *v*_*i*_ being the position and the intensity associated to the *i*th peak or grid point. In the following, it does not make a difference which representation, grid, or peak list eq 1 refers to. For simplicity, we will use the term “peak” to refer to an element of *X* in either case. We define the total weight *V*_*X*_ of a spectrum *X* as the sum of the individual peak weights *v*_*i*_. When comparing two spectra *X* and *Y* of identical total weights, their overall dissimilarity can be quantified by their EMD.^15^ This distance is defined as the minimal “cost” required to redistribute the weight of spectrum *X* so that it resembles *Y*. Here, the cost of transporting weight from point *x* to *y* is computed as the distance *d*(*x*,*y*) of the chemical shift coordinates times the weight that is moved.

**Figure 1:**
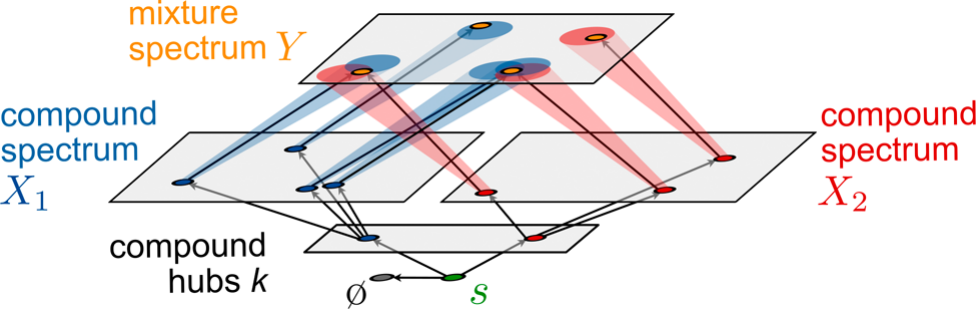
Network architecture of the MCF method. Flow is distributed from the source *s* across the hub nodes *k*, corresponding to individual compounds. Compound hubs distribute flow proportionally to corresponding compound peak nodes that are associated to the compound spectra *X*_*k*_. These are connected to peak nodes of the target spectrum *Y* within an assignment radius *r* (indicated by ellipses in the *Y*-layer). Excess source production is routed to the absorption node *ø*.

When analyzing NMR spectra, the approach of the EMD must be modified. First, the total signal intensity of an NMR spectrum is proportional to the concentration of a sample and we cannot assume *V*_*X*_ = *V*_*Y*_ without losing quantitative information. Second, the EMD allows a nonlocal redistribution of weight, which would correspond to a matching of distant peaks in the NMR context. However, determining whether a peak of a spectrum *X* appears in *Y* requires a locally restricted comparison of intensities. We addressed this problem by introducing an assignment radius *r*. The value of *r* defines the neighborhood of compound peaks *x*_*i*_, in which a matching mixture peak *y*_*j*_ is sought, cf. Figure 1. A detailed description of the network terminology, the relation between our approach and the EMD, the distance used for NMR coordinates, and possible sources of error are provided in the SI (Sections S1–S7).

For the following, we assume that a library  is given, which contains individual compound spectra *X*_*k*_ = {(*v*_*i*_, *x*_*i*_) | *i* ∈ *I*_*k*_}. We use indices from index sets  and *I*_*k*_ to unambiguously refer to elements of the corresponding sets (i.e., a library compound and its peaks). We seek to identify an optimal reconstruction of the target spectrum *Y* as a union of compound spectra scaled by appropriate concentration factors α_*k*_:
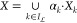
2where α_*k*_·*X*_*k*_ = {(α_*k*_*v*_*i*_, *x*_*i*_) | *i* ∈ *I*_*k*_}. We define the optimality of *X* as the minimality of the costs of a corresponding network flow. The network hosting this flow is constructed as follows (see Figure 1). First, a source node *s* is connected at a specific cost *c*_*ø*_ to an absorption sink *ø* of unrestricted capacity. This means that a flow of volume  running from *s* to *ø* generates a cost . Furthermore, *s* is connected to a layer of hub nodes *k*, which represent the different library compounds. Each *k* is connected to peak nodes *i* ∈ *I*_*k*_ representing the individual peaks of the compound spectrum *X*_*k*_. Finally, each *X*_*k*_-peak node *i* is connected to all nodes representing peaks of the mixture *Y* within the distance *r*, where each *Y*-peak node *j* is equipped with a sink capacity equal to the weight *w*_*j*_ of the peak. The specific cost *c*_*i*→*j*_ of a link connecting *i* ∈ *I*_*k*_ and *j* ∈ *I*_*Y*_ equals the spectral distance *d*(*x*_*i*_,*y*_*j*_) of the peaks. To ensure that assignment to *Y*-nodes is preferred to absorption, we assume that the specific cost of assignment to the absorption sink is larger than the assignment radius, i.e., *c*_*ø*_ > *r*. The flow production at *s* is chosen as  to match the total sink capacity of the target spectrum.

A network flow is defined by the volumes *f*_*n*→*m*_ passing at the links *n* → *m*. We call it “feasible” if it fulfills several constraints:

(i) All production must leave the source:
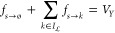
3

(ii) Inflow at *Y*-nodes equals outflow at compound peak nodes:
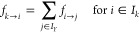
4

(iii) Inflow does not exceed capacity of *Y*-nodes:
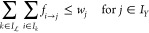
5

(iv) The flow between compound hubs and peak nodes preserves peak proportionality:
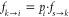
6where *p*_*i*_ = *v*_*i*_/*V*_*X*_*k*__, for *i* ∈ *I*_*k*_.

Any given flow corresponds to a combination (eq 2) of compound spectra, by setting
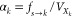
7

Using this, a feasible flow, which minimizes the cost function (i.e., an MCF),
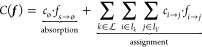
8can be associated to an optimal reconstruction (eq 2) of the mixture spectrum *Y* by means of the compound library .

A Python implementation of the MCF algorithm along with a test suite reproducing the results presented below is available at https://github.com/GeoMetabolomics-ICBM/mcfNMR. The optimization of eq 8 is implemented using HiGHS.^22^

### Datasets

We applied the method described in the previous section to two different sets of experimental ^1^H,^13^C HSQC spectra. The first dataset was published by the Wishart research group, along with their publication of MetaboMiner,^12^ a software suite for semiautomated compound identification. It contains a database of peak lists derived from HSQC spectra of single compounds as well as HSQC NMR data of artificial and biological mixtures, represented either as peak lists or grid data.^23^ The deposited database contains the HSQC chemical shifts of 501 individual compounds and was derived from HMDB.^24^ Please note that the original database contains 502 compounds, but the chemical shifts for lactic acid were deposited twice, which is why we end up with one less entry. We compared the performance of our method with the results reported by Kim et al.,^14^ who compared the performance of their machine learning method (SMART-Miner) among others with MetaboMiner^12^ and COLMAR-HSQC.^13^ As Kim et al.,^14^ we use the artificial mixtures “N925”, “N987”, and “N988”, containing 27, 21, and 24 common metabolites of biofluids in concentrations ranging between 40 and 60 mM, respectively.^12^ We binned the grid data to restrict the resolution resulting in 512 × 512 grid points for “N925” and “N987” and 256 × 512 for “N988”. Furthermore, we tested the MCF method on a plasma sample recorded under different pH (spectra of mixtures “N907” and “N926”, binned to resolutions of 512 × 512 and 512 × 581 datapoints, respectively), which is part of the MetaboMiner dataset, and for which 35 constituents were unambiguously identified.^12^ In addition, we used an in-house data set^100^ with HSQC spectra of 34 individual compounds and different mixtures of these compounds (cf. Section S8 for details) to evaluate the potential of our approach to retrieve individual compound concentrations.

## Results

### Compound Detection

We compared the performance of the MCF method in a compound identification task with three other popular algorithms: MetaboMiner,^12^ COLMAR-HSQC,^13^ and SMART-Miner.^14^ For this benchmark we used a test set of three artificial mixtures and a compound library published by the Wishart lab^23^ containing 501 metabolite spectra.

We tested four different setups A–D of the algorithm (cf. Table 1). Setup A serves as a reference insofar it differs at exactly one aspect from every other setup. The optimization type defines whether the algorithm optimizes the flow for all compounds simultaneously. A simultaneous optimization ensures a consistent reconstruction of the mixture, while for an independent optimization (setup B), we construct the optimal flow separately for each compound. Furthermore, we tested two different variants of flow assignment. The first is the single pass approach described above, and the second uses an incremental version of that approach, which gradually increases the assignment radius (setup C, see Section S7). Finally, in setup D, we compared the results based on grid data (setup A) to those based on peak lists. For an MCF, a compound’s containment is judged based on the comparison of the flow *f*_*s*→*k*_ assigned to this compound and a detection threshold ϑ. The lower ϑ, the more compounds are classified as being contained on the basis of *f*_*s*→*k*_ > ϑ/*V*_*Y*_, where we normalized the detection threshold by the total weight of the target.

**Table 1:** Overview of MCF Method Setups

	optimization	assignment	target
**A**	simultaneous	single pass	grid data
**B**	independent	single pass	grid data
**C**	simultaneous	incremental	grid data
**D**	simultaneous	single pass	peak list

**Figure 2:**
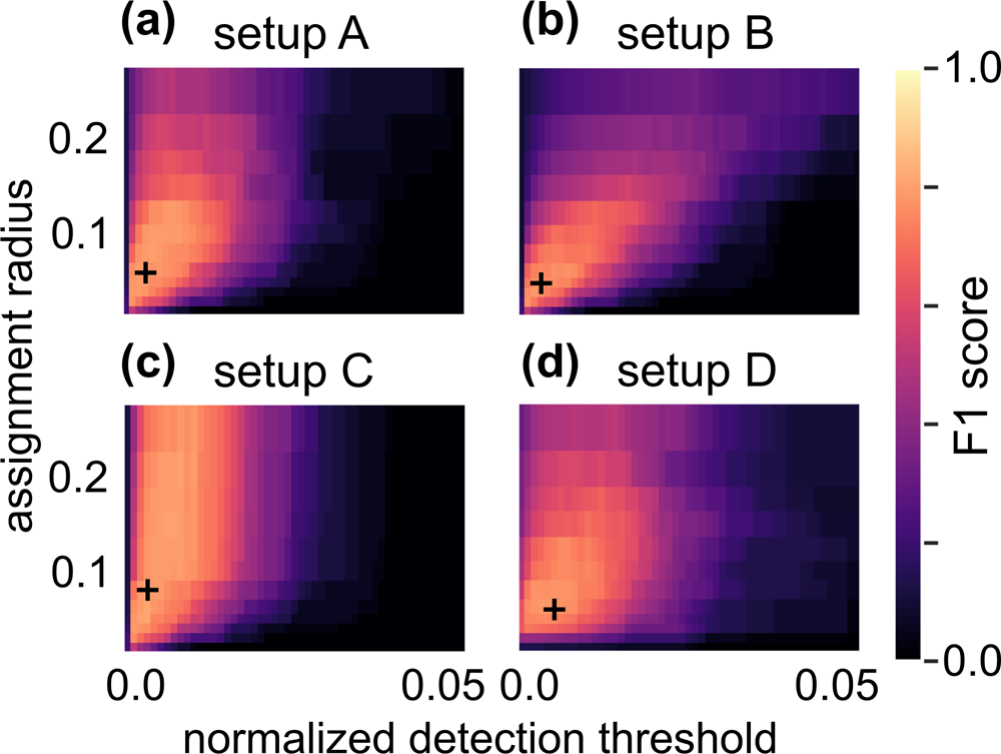
(a–d) Dependence of the average F1 score across mixtures N925, N987, and N988 on the assignment radius *r* and the normalized detection threshold ϑ for the different setups A–D of the MCF method (see Table 1). Markers (“+”) indicate parameter combinations that maximize the average values of the F1 score.

To study the robustness of our method, we scanned a range of combinations of assignment radii *r* and detection thresholds ϑ with a fixed absorption cost *c*_*ø*_ = 10^6^ (see Figure 2). For each combination, we calculated the F1 score from the corresponding values of recall and precision (see Section S9 for details). In general, the best performance (maximal *F*_1_) is achieved for intermediate parameter values. Too low detection thresholds yield many false positives, which decreases the precision. On the other hand, the recall deteriorates for too large detection thresholds. Therefore, both extremes yield low *F*_1_. Similarly, small assignment radii lead to low recall and large assignment radii impair precision. Setup C (Figure 2c) represents an exception to the latter, since the incremental assignment strategy stabilizes the fit with regard to larger radii. As this strategy repeatedly determines MCFs for increasing assignment radii and reserves all flow once assigned, a more precise matching is preferred over a maximal assigned flow volume. As the other setups prefer maximal assignment volume, they exhibit displacement errors for larger radii (see Sections S6 and S7 for details).

The incremental approach (as used in setup C) also achieves the highest average value of *F*_1_ = 0.83 at ϑ = 0.003 and *r* = 0.08. This improves setup A, which only differs in the use of a single pass assignment. There, we found the second best *F*_1_ = 0.81 at ϑ = 0.003 and *r* = 0.06. Slightly worse scores are attained by setups B and D, with *F*_1_ = 0.80 at *r* = 0.05, ϑ = 0.003, and *F*_1_ = 0.78 at *r* = 0.06, ϑ = 0.005, respectively.

Because the maximal value of the performance is not measurable in applications, where it is unknown if a detection is a true or false positive, an important characteristic for each setup is its robustness, with respect to parameter variations. Clearly, the robustness of setup C with respect to *r* is unrivaled. The robustness, with respect to ϑ, is similar for setups A and D being highest for *r* in 0.05–0.1 and decreasing for larger *r*. In contrast, the performance of setup C worsens only slightly for increasing *r* as is reflected in the vertically banded structure of the plot shown in Figure 2c). When fitting compounds independently (setup B), the robustness is significantly worse as *F*_1_ decays more quickly when *r* deviates from its optimum.

**Figure 3:**
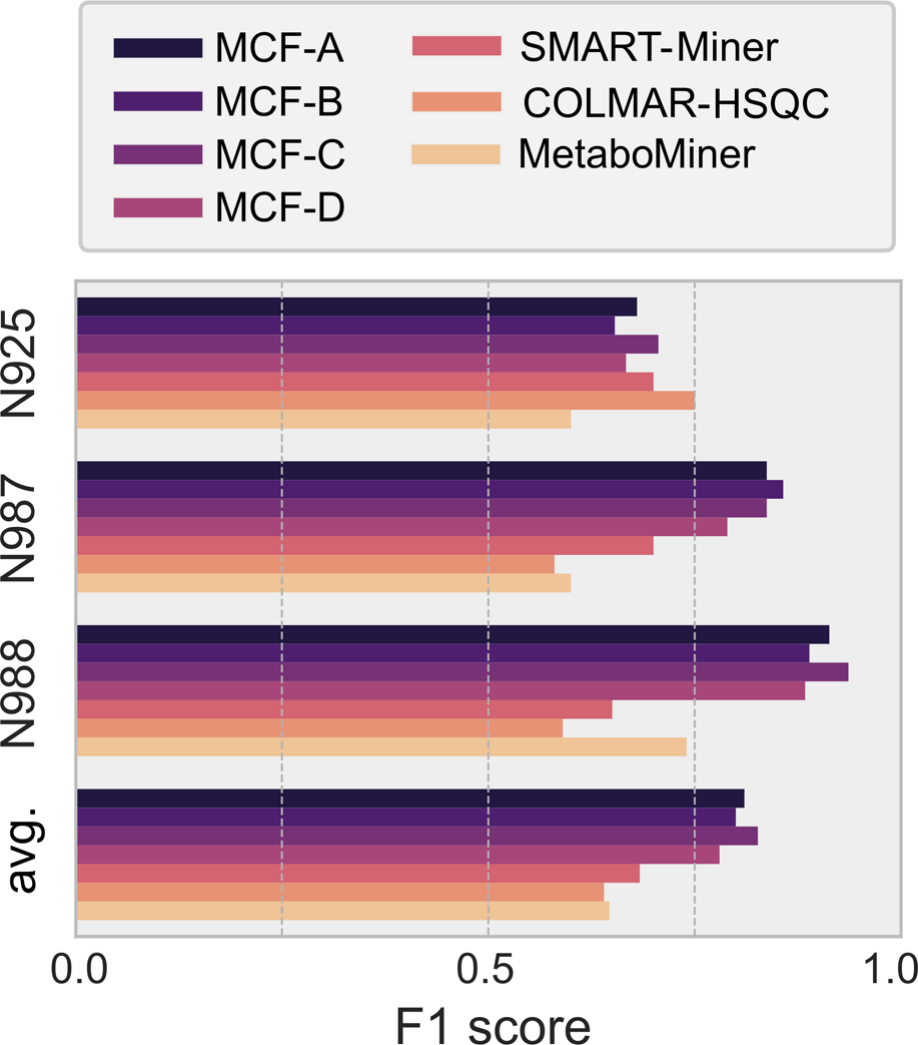
Comparison of F1 scores for different setups of the MCF method (Table 1), and MetaboMiner, COLMAR-HSQC, and SMART-Miner^14^ (Table S3).

When using the combinations of *r* and ϑ leading to the optimal average *F*_1_ across all mixtures, all setups of the flow method outperformed the average performance of all other tested methods by a relative margin of 15% (MetaboMiner: *F*_1_ = 0.65, COLMAR-HSQC: *F*_1_ = 0.64, SMART-Miner: *F*_1_ = 0.68, as calculated from Kim et al.,^14^ cf. Table S3), We compared the attained F1 scores for these parameters and the different mixtures N925, N987, and N988 in Figure 3. For mixtures N987 and N988, all setups A–D display scores *F*_1_ > 0.8 and are clearly superior to other methods. In contrast, for N925, the F1 scores of all methods lie closer together, mostly within the range 0.6–0.7. Only COLMAR-HSQC lies outside that range yielding *F*_1_ = 0.75. We refer to Section S9 for a more detailed analysis, especially of the lower score in the case of mixture N925.

### Application to a Biological Sample

We tested the MCF method on spectra of a human plasma sample recorded under different pH conditions (spectrum N926 at pH 7.3 and spectrum N907 at pH 8.8). Since spectra of library compounds were recorded at physiological pH, a stronger perturbation of peaks can be expected for spectrum N907. Xia et al.^12^ have previously identified 35 compounds in the sample by independent profiling. It is important to note that this list of compounds is not necessarily exhaustive and most likely other compounds are contained in the sample as well. Therefore, we prefer to denote compounds which are detected by a method but not mentioned in the reference list as “unconfirmed” and not as “false positives”. The results should be interpreted with this limitation in mind. For a more detailed discussion, we refer to Section S10 in the SI.

**Figure 4:**
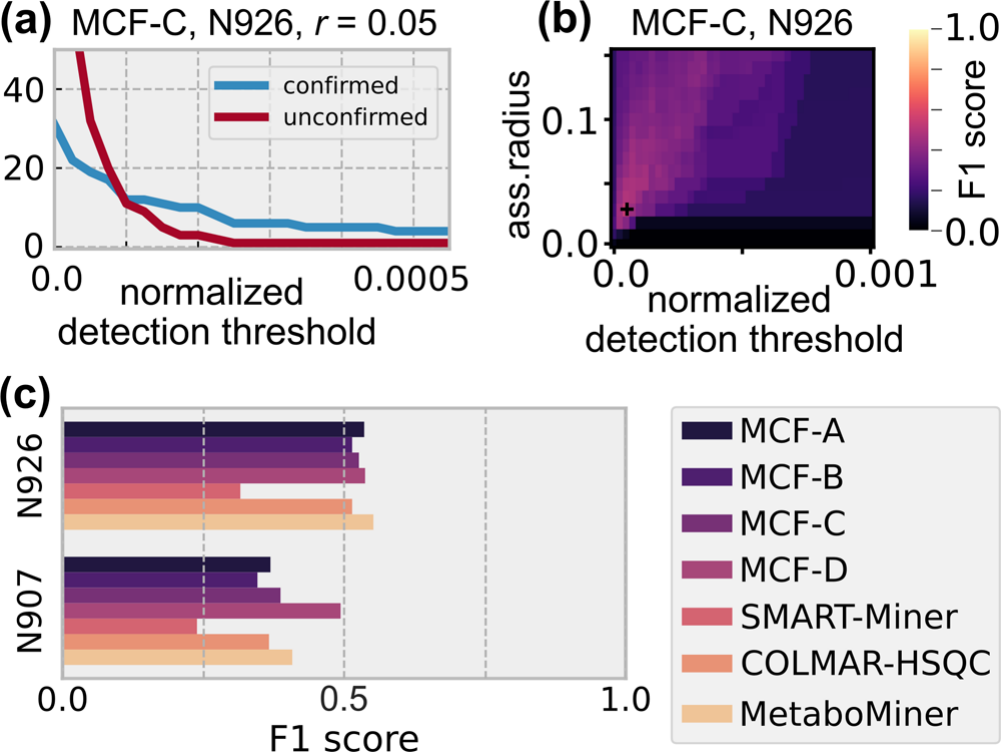
(a) The number of confirmed and unconfirmed detections using the method MCF-C (cf. Table 1) with assignment radius *r* = 0.05 for a plasma sample spectrum (sample N926, from Xia et al.^12^) as a function of the normalized detection threshold. (b) Distribution of the F1 score for a range of different assignment radii and detection thresholds for MCF-C on N926. (c) Optimal F1 scores obtained for different methods on the spectra for samples N926 (pH 7.3) and N907 (pH 8.8).

In Figure 4a, we show the number of compounds detected by the MCF-C method in sample N926, as a function of the detection threshold ϑ for a fixed maximal assignment radius of *r* = 0.05. While a few confirmed compounds are already detected for values of ϑ > 2 × 10^–4^, the bulk remained undetected until ϑ < 10^–4^, where many unconfirmed detections are encountered as well. As for artificial mixtures, recall increases while precision decreases for decreasing values of ϑ. Hence, the distribution of F1 scores exhibits a similar pattern as for the artificial mixtures (cf. Figure 4b and Figure 2) but with significantly lower maximal *F*_1_ = 0.53 at ϑ = 0.5 × 10^–4^ and *r* = 0.03. This greater difficulty to reconstruct the list of confirmed compounds for the plasma sample is common to all tested methods (Figure 4c; also see Table S4 for details). Most methods achieved an F1 score of slightly above 0.5, except for SMART-Miner whose performance dropped most drastically, compared to its results on artificial samples. The altered pH value perturbed the spectral pattern in the case of sample N907 and makes the mixture reconstruction more difficult as testified by the further decreased performances of the different methods. The only exception to this general trend was MCF-D whose performance remained relatively stable, despite these perturbations. In contrast to the other MCF methods, MCF-D operates on peak lists from the MetaboMiner dataset. These were created by automatic peak picking, followed by manual cleaning.^12^ This human intervention seems to be important since automatic peak picking [using DEEP Picker, cf. Table S4, MCF-D (dp)] alone did not lead to significantly better results for MCF-D, compared to other methods.

For comparability with the results reported by Xia et al.,^12^ we also applied our MCF method to the biological samples using the plasma (common) library provided by MetaboMiner. The MCF methods A–C demonstrated similar performance, with F1 scores ranging from 81.6 to 83.5 for sample N907 and 81.6 for sample N926 (cf. Table S5). Compared to MetaboMiner, we observed only a slight performance decrease for sample N926 (81.6 vs. 82.9), but a significant improvement for sample N907 (83.5 vs. 76.2). These findings suggest that the MCF approach could be particularly useful when dealing with altered sample matrices, such as pH variations, which can lead to changes in chemical shift positions.

### Compound Quantification

Besides the comparison with existing algorithms, we have tested the potential of the MCF method to retrieve individual compound concentrations. To this end, we generated optimal reconstructions for different experimental mixtures. All mixtures contained subsets of 34 compounds (cf. Section S8) covering different compound classes, such as amino acids, sugars, and aromatic carboxylic acids. For each compound *k*, we use the following notation:

- *c*_*k*_^°^: compound concentration of the corresponding library spectrum,
- *c*_k_^*^: compound concentration in the target mixture, and
- *c*_*k*_: compound concentration estimated by the MCF method.

**Figure 5:**
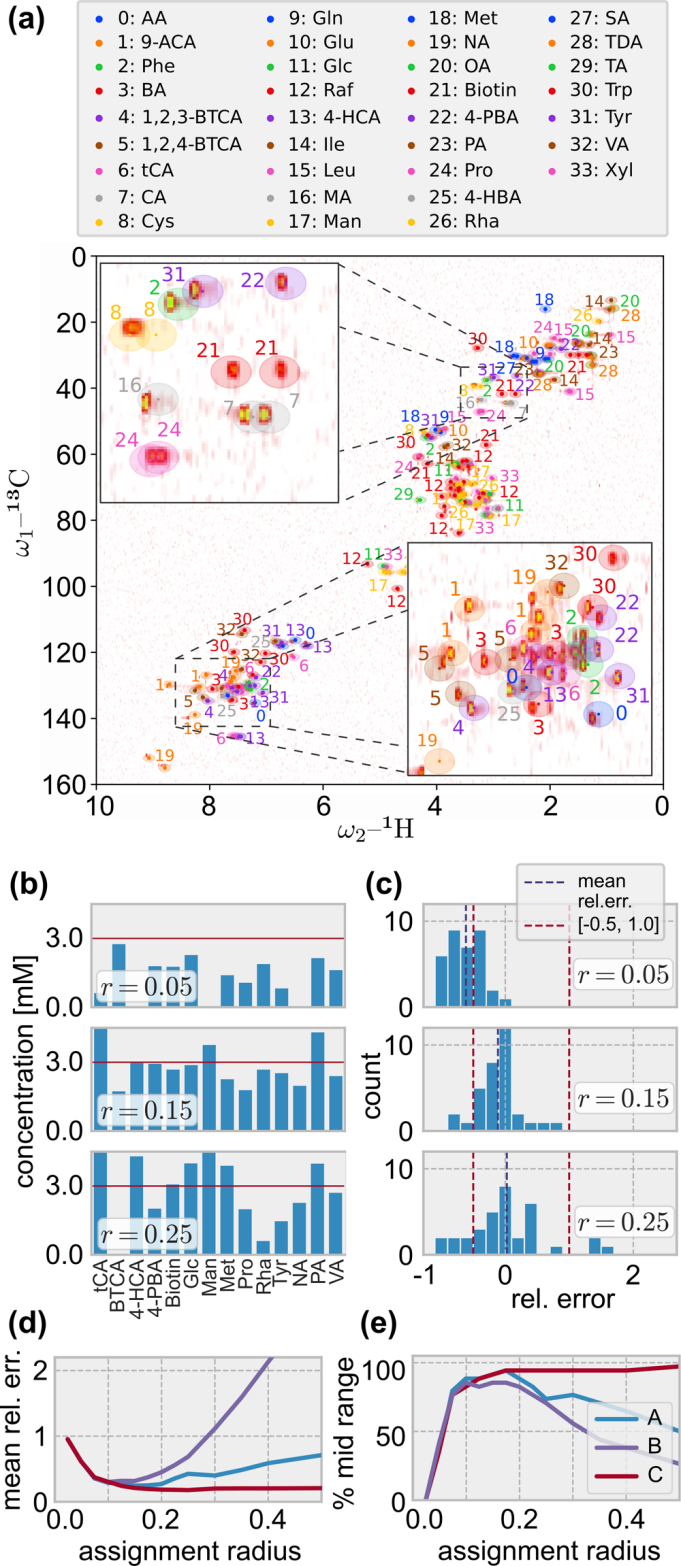
Reconstruction of individual compound concentrations. (a) Overlay of individual compound peaks (ellipses corresponds to *r* = 0.1) on the mixture spectrum, cf. Section S8. (b) Predicted concentrations at different *r* for 14 randomly selected compounds of a mixture containing each compound at a concentration of 3 mM using setup A, *cf*. Table 1. (c) Corresponding distribution of the relative prediction errors. (d) Dependence of the mean relative error magnitude *e̅*, and (e) percentage of errors in the range [−0.5, 1.0] on *r* for setups A–C.

The predicted concentrations in the mixture are calculated as *c*_*k*_ = α_*k*_*c*_*k*_^°^, where the concentration factors α_*k*_ are computed as described in eq 7. Exemplary results are shown for a mixture containing all 34 compounds at a concentration of *c*_k_^*^ = 3 mM. Its ^1^H,^13^C HSQC spectrum constitutes the background of Figure 5a, where peaks of individual compound spectra are shown as overlays. For the quantification, we used grid data for the target spectra and peak lists for the individual compound spectra, which were acquired at concentrations of *c*_*k*_^°^ = 30 mM (cf. Section S8).

Figure 5b compares the true and predicted concentrations for different assignment radii and a subset of compounds obtained for setup A (Table 1). A larger radius permits a larger total assigned flow, because the fraction of the target nodes that are coupled to compound nodes grows with the radius. Thus, the total predicted concentration increases with radius. This does not necessarily hold at the level of individual compounds, though. For instance, the estimated concentration of L-rhamnose (Rha) decreases from *r* = 0.15 to *r* = 0.25. At increased radii, the flow assignments to different compound peaks interfere more strongly, which may lead to such displacements.

In Figure 5c, we show the distribution of the relative prediction errors *e*_*k*_ = (*c*_*k*_ – *c*_k_^*^)/*c*_k_^*^ for different assignment radii. The general trend toward larger amounts of assigned flows improves the fit for radii up to *r* = 0.15. For instance, at *r* = 0.05 many concentrations are underestimated: ∼65% are estimated to less than one-half of the true concentration, cf. left red dashed line at *e*_*k*_ = −0.5. The minimum mean relative error magnitude *e̅* = 0.25 is obtained at *r* = 0.15. That is, on average, the method exhibits a prediction error of 25%. For larger *r*, the prediction becomes worse. For instance, at *r* = 0.25, several compounds display a strong overestimation with *e*_*k*_ > 1, i.e., predicted concentration doubles the true value (see left red dashed line at *e*_*k*_ = 1). The overestimation is related to compound displacements, which can be avoided to a certain degree by using an incremental assignment. This is illustrated by FigureS 5d and 5e, where we show the dependence of the mean relative error magnitude and the percentage of errors within the interval [−0.5, 1.0] on *r* for the different setups. We found that the incremental assignment (setup C) resulted in the most accurate and robust results for this mixture. It optimally reconstructs concentrations at assignment radii around *r* = 0.25 (*e̅* = 0.18). Independent compound optimization (setup B) achieved *e̅* = 0.30 at *r* = 0.1 and is the least robust among the tested setups. For an assessment of mixtures with different compositions of compounds (see Section S11).

## Discussion

Our results indicate a potential for optimal flow methods for NMR-based compound identification and quantification in complex mixtures. For a data set of artificial mixtures used by various authors to validate their algorithms, our method achieved higher F1 scores than all other methods (by a relative margin of 15%). When applied to a natural sample of human blood plasma, most tested methods performed similarly. They achieved F1 scores of values slightly larger than 0.5, pointing toward a potential for further refinements. Finally, our method could predict individual compound concentrations in a mixture of 34 compounds with a mean relative error of less than 20%.

### Unique Features

One major advantage of our method is its ability to perform a simultaneous fit of all individual compounds when reconstructing a complex mixture. This avoids a possible overdetection of compounds associated to independent fits. In Section S4 we provide an example for such an error. As a second important feature, flow methods can be applied directly to grid data. From the flow perspective, grid points, just like the peaks in a peak list, are nodes. This allows to circumvent the step of peak picking, which is a necessary preprocessing step for many other algorithms. This step can not only be a potential source of error, but also reduces the amount of information available to the algorithm. For artificial mixtures, the highest F1 scores achieved by our method were indeed attained when using grid data (cf. Figure 2). However, in the case of complex biological samples, a careful peak identification may still improve the result, as indicated by the improved performance of the peak-list-based method (MCF-D) on the plasma sample, especially for the pH-disturbed sample N907 (cf. Figure 4c and Table S4).

### Single Pass and Incremental Assignment

We explored two approaches for flow construction: a single pass MCF optimization and an incremental assignment (cf. Section S7). Using the MCF approach leads to a lower total assignment cost in general. While this may be beneficial when peaks are shifted in a systematic manner, it is prone to permit displacement errors for larger assignment radii. Such errors are avoided by an incremental assignment, which gradually picks the closest target peaks and reserves their capacity. Hence, once chosen, an assignment cannot be displaced. This simplifies the parameter selection as it stabilizes the fit for larger assignment radii. A possible drawback of the incremental assignment is its bias toward compounds, which only show a few peaks, because it is more likely for a few peaks to coincidentally fall into the neighborhood of target peaks than it is for many (cf. Section S6).

Although the incremental strategy appears to be superior to single pass assignment when tested on artificial mixtures, it is important to note that it may still be prone to overestimation of compound presence. In particular, if a mixture contains many compounds, whose spectra are not included in the library, larger values of *r* will inevitably imply extant assignment. Indeed, when applied to a natural plasma sample, the F1 distribution for the incremental strategy (Figure 4b) is less stable against variations of the assignment radius than for the case of artificial mixtures (Figure 2c). Thus, testing single pass assignment conveys important information about the optimal assignment radius for all setups.

### Compound Library and Reliability

The success of a method for compound identification depends on a few general principles. First of all, it is impossible to match a compound’s peak pattern if it is not in the compound library. One might conclude that the larger the library, the better the reconstruction. But the larger the library, the more candidate combinations exist, making the choice of the correct one more difficult. Thus, for a small library the crucial limitation is that it contains all compounds of the mixture, whereas for a large library the accuracy of prediction may deteriorate with the inclusion of additional compounds. A careful thinning of a large compound library can therefore improve the prediction.

In contrary to the test cases for compound detection, where we used a relatively large metabolite library of 501 compounds, we used a library which contained the same 34 compounds as the mixture when testing our method for compound quantification. This is an optimal library coverage and cannot be expected for most applications. The presence of more compounds in the library could perturb the estimates and we would expect that the reconstruction suffered. The exact impact is difficult to predict, and the method worked similarly well (in terms of relative errors) for mixtures with different concentrations and subsets of the 34 included compounds, which is promising for more complex applications (cf. Section S11).

Compound identification frequently fails due to shifted peaks in the target spectrum or an unreliable library, in which, e.g., peaks may not be picked properly. This implies the importance of highly comparable data that can only be acquired by standardized measurements and stable matrix parameters such as pH, solvent, and ionic strength. For compounds naturally existing as multiple diastereomers, we further suggest to deposit the spectra of the individual diastereomers in the database (cf. Section S11). This approach has already been demonstrated to be beneficial for compound identification based on peak matching,^13^ and we expect further improvements for our approach upon implementation.

### Computational Requirements

At the heart of our method lies a linear optimization that computes an optimal assignment of flow from source to sink nodes. The numerical solution can be challenging, because its computation time increases with the number of links in the network. In theory, this increase can be polynomial or even exponential, depending on the algorithm.^20^ However, in practice, we rather observed a roughly quadratic increase in computation time with the number of links in the flow network (cf. Section S12). Since the theory of MCFs and linear programming in general has a long tradition, efficient solvers are available for most problems, which facilitates an implementation of the method. Especially when using grid data, which leads to much larger networks than peak lists, the computation can nevertheless be time- and memory-consuming. For working with grid data, it is important to choose an optimization software that supports parallelizable sparse matrix implementations (for our implementation, we used the SciPy^25^ interface to HiGHS^22^).

### Choice of Parameters

In applications, our method is supposed to reveal the unknown composition of a sample. Before it can do so, the user must select parameter values for the assignment radius *r*, for the absorption cost *c*_*ø*_, and, if a binary classification (contained versus not contained) is desired, for the detection threshold ϑ. Hence, they are confronted with the question for which parameter values the method will give the most accurate answer. Also, it may be more desirable to avoid a specific type of error rather than another and either increase the precision or recall instead of maximizing a synoptic measure such as the F1 score. To date, we have no definite answer or protocol to choose these values. Some ideas to guide the choice are described below.

The assignment radius *r* defines the tolerance to peak position shifts and should therefore usually encompass the expected magnitude of such shifts. If the target spectrum is represented as grid data, *r* should additionally be large enough to capture the entire extension of a peak. Hence, in most cases, a good choice of *r* would be the maximal accepted shift distance plus, for a grid target, the half-width of a peak. The optimal assignment radius may differ for quantitative and qualitative purposes. For quantitative reconstruction, it is important to capture the full peak extension. This can require a larger *r* than for detecting the presence of a compound, where a partial coverage of the peak extension may already suffice. In agreement to this, we found that the optimal *r* (for non-incremental setups) lies around *r* = 0.05 for compound detection (cf. Figure 2) and around *r* = 0.15 for compound quantification (cf. Figures 5d and 5e). This is in accordance with the observed peak widths between 0.03 and 0.09 ppm along the ^1^H dimension in the experimental spectra.

When using incremental flow assignment, the user is relieved from the choice for *r* to some degree as the results becomes more stable (cf. Figure 2c). However, it cannot stabilize the effect of the detection threshold ϑ. A lower threshold yields a larger number of detections and, hence, a higher recall (cf. Figures S6 and S8). However, if the threshold approaches the noise level, the number of false positive detections will usually rise considerably for any method, implying a drop of precision. On the other hand, choosing a high threshold effectively discards fractions of the mixture, which fall below a certain concentration, decreasing the recall. Values from ϑ = 0.005 × *V*_*Y*_ to ϑ = 0.01 × *V*_*Y*_ worked well for the artificial mixtures tested. As these mixtures contain compounds at relatively high concentrations (40–60 mM), more complex samples may require a higher sensitivity to identify more dilute components, as confirmed by our tests using a plasma sample. When fitting a grid target, decreasing ϑ increases the risk of accidentally matching the noisy background and detecting many false negatives. A first guess for an appropriate choice of detection radius may thus be obtained by observing the dependency of the number of detections in a blank spectrum with the same noise characteristics on the threshold value. Then, a reasonable choice for ϑ may be the smallest value for which no compounds are detected in the blank spectrum.

We did not elaborate on the choice for the absorption cost *c*_*ø*_ in this work but used a value (*c*_*ø*_ = 10^6^) exceeding the assignment radius *r* by several orders of magnitude. This choice implies that an optimal flow maximizes the volume of assigned flow, because all unassigned flow creates absorption costs. As illustrated by a simple example in Section S6, this may lead to a bias toward assigning flow to compounds with a higher number of peaks. Only if *c*_*ø*_ is of the same order as *r*, compounds may be preferred if they offer the more precise fit, even if this implies additional flow to be absorbed.

In summary, the suitability of parameters depends on the data – especially on the complexity, the peak shifts, and noise level of the target spectrum – and on the purpose of the analysis, which puts the focus on precision or recall.

### Measuring Confidence

Developing methods for testing the reliability of a reconstruction is beyond the scope of this work, but we see two major directions to pursue here. One is a perturbative approach, which computes several results based on slightly perturbed (library and/or target) spectra and assesses the variability of predicted containment or concentrations under different perturbations. If the prediction for an individual compound is stable under perturbations, the confidence for the results would be higher than for a compound whose prediction strongly depends on small perturbations (cf. Section S5 for a situation giving an unstable fit).

An alternative approach could test the variability of prediction with the underlying library. Similarly as for the perturbative approach, if a compound prediction is persistent under the usage of different subsets of the full library, its confidence would be higher. This approach resembles a classical bootstrapping approach and could also reveal dependencies between different compounds within the library. Since such dependencies arise due to competing assignments to target sinks, a similarity analysis of the library might reveal them. In particular, a cluster analysis can help to identify groups of compounds, which are most likely to influence each other’s predictions.

Other authors developed heuristics pointing in this direction. For instance, Xia et al.^12^ counted the number of neighboring peaks for each peak within the compound spectra library and Bingol et al.^13^ counted the number of uniquely assigned compound peaks for each peak of the target spectrum. Both approaches give measures of compound interference at a given peak and may therefore serve as indicators for the stability of the peak assignment under library subsampling.

### Final Remarks

While the MCF method shows promising potential for analyzing two-dimensional (2D) NMR spectra, its current implementation faces several challenges and limitations: (i) reliance on choice of parameters and method variant, (ii) sensitivity toward peak shifts, (iii) diminishing performance for mixtures of increasing complexity, (iv) limited quantification accuracy, and (v) peak picking may outperform direct application to grid data in specific scenarios. Many of these points are common to other methods. To conclude on the robustness, we further suggest to conduct an in-depth statistical assessment of key variables influencing performance, such as mixture complexity, library size, noise, dynamic range, and matrix effects.

## Conclusion

Novel flow-based methods can boost the performance for certain classification tasks and offer the potential to estimate individual compound concentrations. They are agnostic with respect to peaks, but believe in nodes. A node is not required to be a local maximum of a distribution, but only a point in spectral coordinates carrying a specific weight. This could open up a way to analyze very complex mixtures, whose spectra rather consist of broad intensity distributions than of defined peaks. In such cases, methods that rely on peak picking are facing a fundamental obstacle, while flow methods still provide an optimal reconstruction using a library of individual compounds. When moving from metabolomic applications to the analysis of highly complex environmental samples such as dissolved organic matter,^26^ this represents a decisive advantage. In such samples individual peaks often cannot be resolved, even when using high-field NMR instruments.^27,28^ Nevertheless, an MCF reconstruction may still allow to derive details of the distribution of compound classes, which is already a valuable information for many environmental problems.

For spectra with pronounced peaks, a better integration of peak shapes when fitting grid targets may be a valuable extension of the algorithm. If implemented successfully, such approaches could integrate a more precise reconstruction of overlapping peaks currently addressed separately, e.g., by deconvolution approaches such as DEEP picker^7^ or by manual inspection. Additional post-processing of the target spectrum (e.g., noise and streak removal) can be expected to further improve the performance. Another promising extension of the basic MCF method is the introduction of tolerance toward missing peaks. This can be achieved by introducing penalties for unmatched peaks rather than excluding the whole compound.

Regarding compound quantification, we await the relevance of the offered capabilities to increase as technological facilities for high-quality NMR measurements are becoming more accessible. Technological advances will yield a larger body of data and more opportunities for the application of concentration recovery, which needs a precise standardization to ensure interoperability of the efforts of different laboratories.

## Supporting Information Available

Additional information on flow methods, network terminology, illustrative examples and detailed information on reconstruction experiments (PDF)
