# Supplementary material for: Network Flow Methods for NMR-Based Compound Identification
Source: Anal Chem. 2025 Feb 25;97(9):4832–40. doi: 10.1021/acs.analchem.4c01652 (PMC11912116; doi:10.1021/acs.analchem.4c01652)
Supplement: Supplementary file 1 — ac4c01652_si_001.pdf [file ac4c01652_si_001.pdf]

Supporting Information

Network Flow Methods for NMR-Based Compound Identification

Leonhard Lücken,[*,†,¶] Nico Mitschke,[*,†,¶] Thorsten Dittmar,[†,‡] and Bernd Blasius[†,‡]

†*Institute for Chemistry and Biology of the Marine Environment (ICBM), Carl von Ossietzky Universität Oldenburg, Ammerländer Heerstraße 114-118, 26129 Oldenburg, Germany.*

‡*Helmholtz Institute for Functional Marine Biodiversity, Carl von Ossietzky Universität Oldenburg, Ammerländer Heerstraße 114-118, 26129 Oldenburg, Germany*

¶*L.L. and N.M. contributed equally to this work.*

* E-mail: leonhard.luecken@uol.de

* Email nico.mitschke@uol.de

Contents

S1 Network Flow Glossary **S3**

S2 Earth Mover's Distance for NMR Spectrum Comparison **S5**

S3 Assignment Radius and Maximal Embedding **S6**

S4 Inconsistent Indication **S7**

S5 Linear Dependence **S8**

S6 Sources of Bias **S9**

S7 Incremental Reconstruction **S11**

S8 In-House Dataset **S13**

S9 Supplementary Results: Compound Detection **S15**

S10 Supplementary Results: Plasma Sample **S19**

S11 Supplementary Results: Quantification **S24**

S12 Computational Requirements **S28**

References **S29**

S1 Network Flow Glossary

Term	Definition	In context of the flow method
network	A set of nodes with links between them.	A network designed to calculate an optimal association of intensities between different spectra to calculate their similarity or to determine the optimal reconstruction of a target spectrum.
node	Basic elements of a network, which produce a defined amount of flow or hold a defined sink capacity for flow.	Most nodes represent peaks (or intensity data on a grid point) of spectra (of single compounds or mixtures). For complex mixture reconstruction, additional nodes are compound hubs, global source and absorption node. Nodes corresponding to peaks of the target spectrum are sinks. For the EMD network, the peak nodes of the source spectrum are sources. For the reconstruction and embedding networks, the only source node is the global source.
link	Connects two nodes. In a directed network it has a source and a target.	For setups using an assignment radius r, source or compound peak nodes are connected with all target peak nodes, which lie within a distance r.
sink	Node that has a positive sink capacity.	Sinks in our setup are the nodes associated to peaks of the target spectrum.
source	Node that has a positive production.	For the EMD setup, nodes of one spectrum are designated as sources. For other setups, we use a specific global source node.
network flow	A set of values associated to links, representing flow passing from the sources to the targets of these.	Represents redistribution of the intensity of the source spectrum or of a global source to the target peak nodes.
absorption node	Sink with unrestricted capacity, which absorbs all flow that cannot be assigned to other sinks.	The sink for all flow, which is not assigned to target peak nodes.
specific cost	Costs associated to a link that are caused when flow passes the link. The specific cost is the cost per unit flow.	The specific cost of a link between two peak nodes corresponds to their distance in the spectral coordinate system.
flow cost	For a given network flow, its cost is the specific link cost times the flow passing the link summed up over all links.	The product of the amount of spectral intensity and the distance, over which it is transported.

**Term**	**Definition**	**In context of the flow method**
minimum cost flow	The flow that minimizes the total flow cost among all feasible flows.	Gives the EMD for the EMD setup and defines a notion of optimality for mixture reconstruction from single compounds in the reconstruction setup.
sink capacity	The amount of flow, which may enter a sink without leaving it again.	Corresponds to the intensity associated to the target peak node, which is represented by the sink.
source production	A defined amount of flow produced in a source. Under a feasible network flow it leaves the node.	For the EMD setup, this corresponds to the intensity associated to the source peak node. For the setups using a global source, it is the flow produced there and should be large enough to eventually serve all sink capacities in the target peak nodes, i.e., the total intensity of the target spectrum.
global source	The source in networks with a single source.	The unique source of intensity flow, from which flow is directed either to the compound hubs or the absorption node.
absorption	Flow running into the absorption node.	Produced flow, which is not distributed to the target peak nodes must leave the source and is absorbed.
hub	A node connected to many others.	We call a class of nodes compound hubs, because they distribute all incoming flow to the peak nodes corresponding to a compound. The outgoing flows have the same proportionality as the compound peak intensities.

S2 Earth Mover's Distance for NMR Spectrum Comparison

The Earth Mover's Distance (EMD) measures the distance of distributions over a given domain and was originally derived as a solution to the problem of distributing economic goods as efficiently as possible[1,2]. It is now used across a wide variety of fields, such as image retrieval[3], medical diagnosis[4], oceanography[5], and ecology[6].

The EMD of spectra X and Y [*cf.* Eqn. (1) of the main text] can be obtained as a minimal cost flow (MCF) on a directed network[7]. The nodes of this network correspond to the peaks (v_i, x_i) and (w_i, y_i) of X and Y, respectively. An X-node i is a source and assumed to produce an amount v_i of flow that has to be transported to Y-nodes, which are sinks and can take up an amount of flow corresponding to the associated weights w_i.

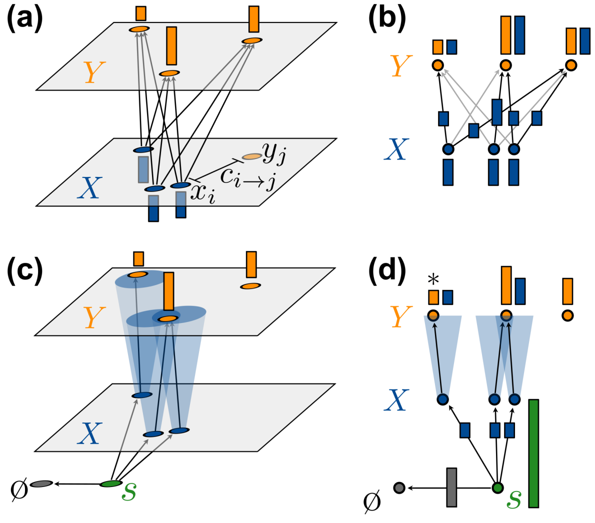

Figure S1: EMD and maximal r-embedding as MCF. Nodes are indicated by discs and arrows represent the links connecting sources to sinks. Bars next to the nodes indicate the corresponding sink capacity or source production. (a) Fully linked flow network for the calculation of the EMD of two 2D spectra X (blue) and Y (orange). (b) Schematic 1D representation of the MCF corresponding to the EMD of X and Y. Bars on links indicate the amount of flow passing the link. Blue bars at the Y-layer indicate the amount of flow reaching the corresponding sink. (c) Setup to compute a maximal r-embedding with assignment radius r, source s and absorption node ϕ. (d) MCF corresponding to a maximal r-embedding of X in Y. The asterisk $*$ indicates the limiting target sink capacity.

In Figure S1(a) we illustrate this with a simple example. An MCF is an assignment of flows $\boldsymbol{f} = \{f_{i\to j}\}$ between all nodes i of X and j of Y, which transfers all production of X into Y and minimizes the total cost

$$C(\boldsymbol{f}) = \sum_{i\in I_X}\sum_{j\in I_Y} c_{i\to j} f_{i\to j}, \tag{S1}$$

with $c_{i\to j} = d(x_i, y_j)$. The condition, that the flow should transport all production in X to sinks in Y respecting their capacities is

$$\sum_{j=1}^{n_Y} f_{i\to j} = v_i, \text{ and } \sum_{i=1}^{n_X} f_{i\to j} \le w_j. \tag{S2}$$

The minimum cost flow for our example is shown in Fig. S1(b). In the example, we assumed that the total weight of X,

$$V_X = \sum_{i=1}^{n_X} v_i, \tag{S3}$$

equals the total weight $V_Y = \sum_{i=1}^{n_Y} w_j$ of Y. Therefore, the sink capacities w_j are filled completely.

S3 Assignment Radius and Maximal Embedding

In the main text, we introduced the notion of an assignment radius r to achieve a more local matching of weight of X and Y than in an unrestricted EMD. This is implemented by cutting all links between nodes corresponding to peaks with $d(x_i, y_j) > r$ in the EMD network. Here we construct a notion, which lies one step in between the EMD network (see SI Sec. S2) and the mixture reconstruction network with multiple compound hub nodes (see main text, Fig. 1). The restriction of linkage may impede that all production, i.e. V_X, can be assigned to matching Y-sinks (for instance, if a peak in X has no corresponding Y-peak at all in its r-neighborhood). We call X r-embeddable into Y, if a flow that satisfies (S2) exists on a restricted EMD network, where all links for nodes with $d(x_i, y_j) > r$ have been removed. Such a flow is called an r-embedding.

Even if X is not r-embeddable into Y, we may still ask: How much of it is? To answer this, we seek a maximal factor α, such that for $\alpha X = \{(\alpha v_i, x_i)\}$ an r-embedding into Y exists. This embedding is called maximal. Similar as for the EMD, we obtain the maximal embedding as a minimum cost flow. To achieve this, we add two special nodes: (i) the global source s, endowed with a weight $v_s = V_Y$, hence large enough to eventually serve all sinks, and (ii) an absorption sink ϕ with unrestricted capacity, *cf.* Fig. S1(c). The source node is connected to the X-nodes at no cost and to the absorption node at an absorption cost c_ϕ. This cost should be chosen larger than the assignment radius ($c_\phi > r$), because this yields a preferential assignment of flow to Y-nodes. Further, X-nodes do not carry any production

by themselves in this setup but exclusively receive an inflow $f_{s\to i}$ from the global source node s. In order to represent an embedding of αX, this inflow must preserve the proportional peak weights of X, i.e., $f_{s\to i} = \alpha v_i$. This is achieved by the additional set of constraints

$$f_{s\to i} = p_i f_{s\to X}, \tag{S4}$$

with $f_{s\to X} = \sum_{i\in I_X} f_{s\to x_i}$ and $p_i = v_i/V_X$. The flow $\boldsymbol{f}$ fulfilling (S4) and

$$f_{s\to\phi} + \sum_{i\in I_X} f_{s\to i} = V_Y, \text{ and, } \sum_{j\in I_Y} f_{i\to j} = f_{s\to i}, \tag{S5}$$

and the capacity constraints

$$\sum_{i=1}^{n_X} f_{i\to j} \leq w_j,$$

where $f_{i\to j} = 0$ if the link is not part of the restricted network. Minimizing the costs

$$C(\boldsymbol{f}) = \underbrace{c_\phi f_{s\to\phi}}_{\text{absorption}} + \underbrace{\sum_{i\in I_X}\sum_{j\in I_Y} c_{i\to j} f_{i\to j}}_{\text{assignment}}, \tag{S6}$$

yields a maximal embedding $X^* = \{(f_{s\to i}, x_i)\} = \alpha^* X$ with $\alpha^* = f_{s\to X}/V_X$. Figure S1(d) shows the maximal r-embedding for X and Y from the previous example. As all peaks in X carry the same weight, the flow through each X-node is the same, that is $p_i = 1/3$ in (S4), since X has three peaks. In contrast to the EMD [in Panel (b)], only one sink (marked with an asterisk) is fully exhausted. This forbids any further assignment of flow to other sinks, since the proportionality constraints (S4) would require more flow to be assigned to the exhausted sink as well.

We remark that the optimal mixture reconstructions $X = \cup_{k\in I_\mathcal{L}} \alpha_k X_k$ mentioned in the main text all represent maximal r-embeddings for the corresponding assignment radius r. And even more strictly: each α_k should be maximal in the sense that for any increase, the resulting combined spectrum cannot be embedded in Y.

S4 Inconsistent Indication

A simplified problem of compound identification is shown in Fig. S2(a). Here, the target Y contains four peaks of identical weight: $Y = \{(w, y_i) \mid i = 1, ..., 4\}$. Two candidate compounds with spectral representations X_1 and X_2 (both represented by three peaks) are given, whose peaks lie close (at distance $< r$) to peaks of Y and have unit weights as well. Hence,

both compounds considered independently should yield a positive indication for their containment. However, a spectral overlap exists between X_1 and X_2, i.e., two X_1-peaks lie close to two X_2-peaks. This introduces a dependency: fitting more of X_1 should leave less capacity for X_2 and *vice versa*. An independent fit cannot capture such interactions adequately. If a hypothetical reconstruction of Y in the form

$$Y \approx (\alpha_1 X_1) \cup (\alpha_2 X_2) \,. \tag{S7}$$

was derived from individual compound fits it would exceed the true peak weights at y_3 and y_4, see Fig. S2(b). A better solution would perhaps associate the Y-peaks $j = 1, 3$, and 4 exclusively to X_1, as its peaks are on average closer to Y than those of X_2. This would leave the peak at y_2 unexplained, hence indicating an unknown component.

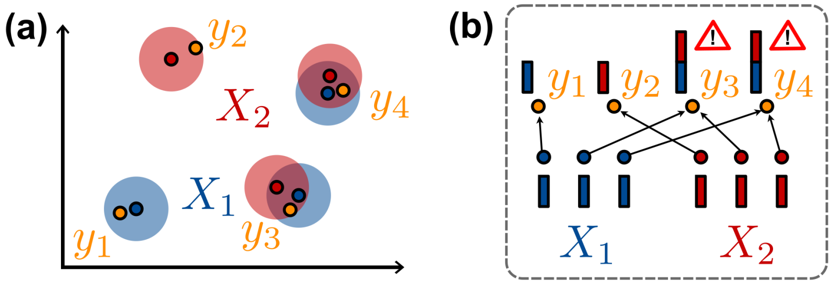

Figure S2: Inconsistent assignment when fitting compounds individually. (a) Overlay of compound spectra X_1 (blue), X_2 (red), and target spectrum Y (orange). Assignment radii are indicated by shaded discs. (b) Schematic representation of the composition $X_1 \cup X_2$ whose combined assignment exceeds the weights in peaks y_3 and y_4. The best consistent fit would only indicate the containment of X_1.

S5 Linear Dependence

A fundamental problem for compound identifiability, which may not be completely resolvable, is the possible linear dependence of compound spectra. This becomes apparent if we assume that two different combinations of compound spectra can equally well explain the target Y. Figure S3 illustrates a case, where a reconstruction of Y is equally good by combining either X_1 and X_2, or X_3 and X_4. It is impossible to decide, whether $X_1 \cup X_2$, or $X_3 \cup X_4$, or a combination of all four compounds is the better reconstruction of Y. It may be objected that in reality a strict linear dependence may not be expected, since the space of peak patterns has infinite dimension. However, if the average distances of the different combinations (for this example X_1 and X_2, or X_3 and X_4 respectively) from Y are similar,

any methodical decision for such a combination is unstable to relatively small perturbations in the peak coordinates. If Y-peaks are modeled with spatial extension (i.e., for grid data), the situation would lead to a combination of all four spectra, which may or may not reflect the truth.

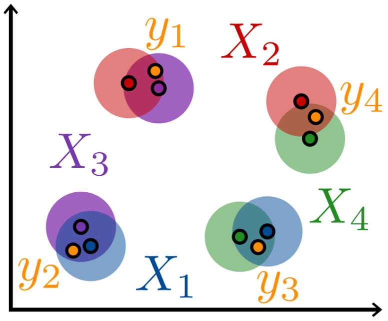

Figure S3: Linear dependence of library compounds: Overlay of four compound spectra (red, green, purple, and blue) which allow different combinations to explain the target spectrum (orange).

S6 Sources of Bias

Simultaneous, flow-based fitting of all compounds avoids errors leading to over-assignment by taking into account the overlap of individual compound patterns (*cf.* Fig. S4). Such an overlap may introduce a "competition" for source capacity between different compounds, which improves the reconstruction in most cases but can also err. For instance, consider a library of two compound spectra, $X_1 = \{(w, x_1)\}$ and $X_2 = \{(w, x_2), (w, x_3)\}$, and a target spectrum $Y = \{(w, y_1), (w, y_2), (w, y_3)\}$, *cf.* Fig S4(a). For simplicity, we assume that all peaks (of all X_k and Y) have identical weight w. X_1 has a single peak within a small radius (dark blue disc) to Y, whereas X_2 has none. Only for a larger radius (lighter red discs), both peaks of X_2 have neighboring points in Y within that range. This may either occur because peaks are perturbed due to unstable measurement conditions or matrix effects or because compound 2 (corresponding to spectrum X_2) is not really part of the mixture. However, which compound is indicated by the minimum cost flow method depends on the assignment radius r and the absorption cost c_ϕ. If a small assignment radius is used, *cf.* Fig. S4(b), X_2 is not r-embeddable. Therefore, all non-absorbed assignment will flow from the source node into the hub node for X_1, further into the single peak node for x_1, and ultimately into the peak y_1, see Fig. 1 of the main text. Since y_1 has a capacity of w and the total weight of Y is $V_Y = 3w$, a volume of weight $2w$ will be absorbed into node ϕ. This yields a total cost of

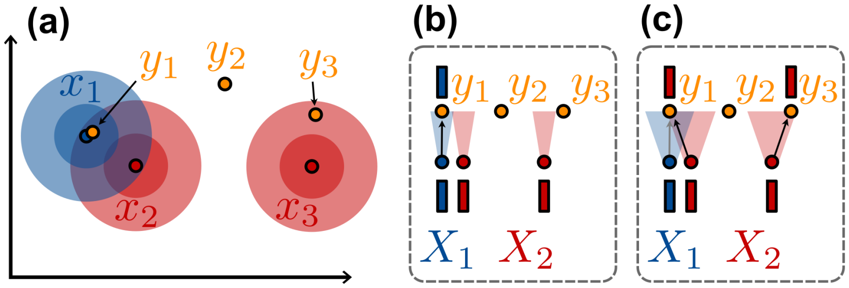

Figure S4: Example for assignment biases when fitting two compounds. (a) Overlay of compound spectra X_1 (blue), X_2 (red), and target spectrum Y (orange). Shaded circles indicate different assignment radii (dark shading: smaller r, light shading: larger r). (b) Optimal assignment for small r selects X_1. (c) For larger r, compound X_2 with more peaks is preferred.

$C_1 = w \cdot c_1 + 2w \cdot c_\phi$, where $c_1 = |x_1 - y_1|$. For a larger assignment radius, *cf.* Fig. S4(c), an embedding of X_2 becomes possible. Since X_1 and X_2 compete for y_1, the minimum cost flow will either choose X_1 or X_2. As X_2 has two peaks, a total assignment of $2w$ is possible for this variant and a volume w is absorbed. The corresponding cost is $C_2 = 2w \cdot \bar{c}_2 + w \cdot c_\phi$, where $\bar{c}_2 = (|x_2 - y_1| + |x_3 - y_3|)/2$ is the specific cost for assignment to compound 2. If the absorption cost is high, the minimum cost flow is the one leading to the lowest amount of absorption. In our example, this is the assignment to X_2. For lower absorption cost, the algorithm may still favor X_1. But this is only true for a considerable mismatch of X_2 (i.e., specific costs $\bar{c}_2$ on the order of r). Hence, there is a general tendency of the method to favor compounds with many peaks, especially for larger values of r and c_ϕ. This may thus lead to displacement of compounds with less peaks even if their peaks match peaks in the target spectrum more precisely.

At the same time, an opposite type of bias exists, which favors compounds with fewer peaks. It may arise if r is small, which may lead to a false negative if the displacement of a compound's peak is larger than r. In that case, at least one peak becomes disconnected in the network and no assignment to the compound node h_k is possible. This is more likely if a compound has many peaks. Therefore, the chance for a compound to be dismissed may also rise with the number of its peaks. Which of these two effects is stronger in a specific application is difficult to predict. In general, we expect a larger assignment radius to rather imply a bias towards compounds with spectra containing many peaks.

S7 Incremental Reconstruction

S7.1 Incrementing the Assignment Radius

An incremental variant of the MCF-based algorithm can overcome the bias towards compounds with higher numbers of peaks and stabilize the fit for higher assignment radii. It uses the same network as the single pass MCF calculation (see Fig. 1 of the main text) and follows a greedy assignment with gradually increasing the assignment radius r to arrive at a feasible flow, *cf.* Eqn. (3)–(6) of the main text. That is, as the assignment radius is increased, source production is incrementally assigned to the best-matching compound and flow once assigned is preserved.

This is illustrated for an example in Fig. S5, which shows the assignment of two compound spectra $X_1 = \{(w, x_1)\}$ and $X_2 = \{(w, x_2), (w, x_3)\}$ to a target spectrum $Y = \{(w, y_1), (w, y_2), (w, y_3)\}$ for a series of assignment radii. For the smallest radius $r = r_1$ [panel (a)], no flow can be assigned through neither X_1 nor X_2 because no target peaks are within distance r_1 of the compound peaks. The next larger radius r_2 [panel (b)] allows for assignment through X_2 because nodes corresponding to target peaks y_1 and y_2 are now connected to the X_2 nodes. The assignment is indicated by the red edgecolor of markers for y_1 and y_2. At this stage there is still no difference between the single pass and incremental assignment strategy. But as the radius is further increased until $r = r_3$, such that y_1 is within assignment distance of X_1 [panels (c) and (d)], substantial differences for incremental assignments arises compared to single pass assignment. Assuming that the absorption cost c_ϕ is relatively high, the single pass optimization [see Fig. S5(d)] will result in a flow, which assigns as much flow as possible (to avoid expensive absorption). This leads to an assignment $x_1 \to y_1$ for X_1 and $x_2 \to y_2$, $x_3 \to y_3$ for X_2 (indicated by edgecolors of the Y-markers). For an incremental assignment, the flow assigned in the previous steps (here, only at $r = r_2$) is reserved. Even though y_1 is in reach of x_1, in this case the sink capacity of the node corresponding to y_1 is already exhausted and no further assignment is possible. Only if the radius is increased further to $r_4 = d(x_1, y_3)$ an assignment to x_1 will occur following the incremental algorithm.

For definiteness, let us consider how the incremental assignment can be constructed in general. This can be achieved in discrete steps, where, at the n-th step we denote the current radius by r_n and the remaining sink capacities by $w_{j,n}$, $j \in I_Y$, at the target peak nodes. Starting with a radius r_0 equal to the the minimal distance of a compound peak to a target peak, and sink capacities $w_{j,0} = w_j$, we connect all compound and target peak nodes within that radius r_n and calculate the corresponding MCF $\boldsymbol{f}_n$. For the next step, we set r_{n+1} to the distance of the next closest compound and target peak nodes not yet connected. Further, we

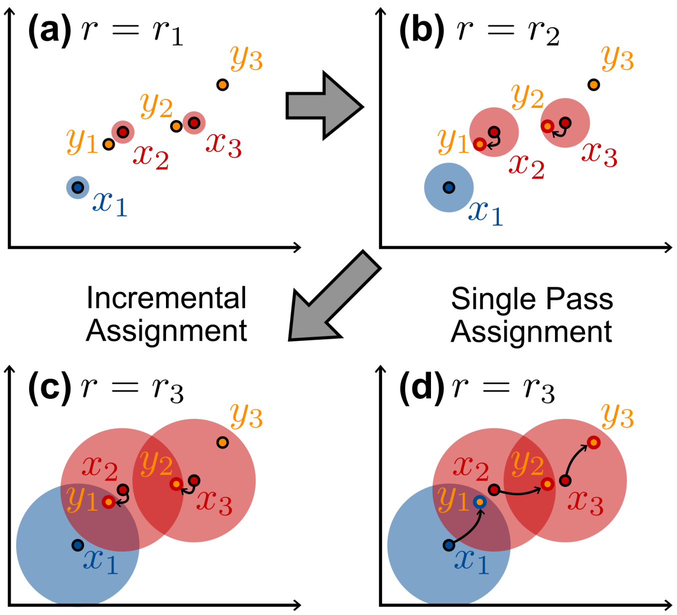

Figure S5: Different assignments for increasing radii $r_1 < r_2 < r_3$ and different strategies (incremental radius and single pass). Shaded circles indicate different assignment radii. Peaks of target spectrum Y (orange) and of the compound spectra X_1 (blue) and X_2 (red) are indicated as dots. The peak matching induced by the corresponding strategy is indicated by edgecolors of the Y-peak dots and small arrows. Thick, gray arrows indicate the incremental sequence finally leading to the assignment (c). For r_1 and r_2 both strategies yield the same result. The differing single pass result for r_3 is shown in (d).

decrease the sink capacities $w_{j,n}$ by the flow assigned to them via $\boldsymbol{f}_n$ to obtain $w_{j,n+1}$. This is repeated until a defined maximal assignment radius r is reached. Practically, the iteration can be implemented by steps of discrete radius increments. This may add multiple new connections with different costs at each step. Although this can lead to displacements (*cf.* Sec. S6), the resulting flow approximates the one obtained by adding connections one-by-one for small radius increments.

S7.2 Incrementing the Source Production

A similar algorithm can be obtained by gradually increasing the source production. Consider the situation that an infinitesimally small quantum of source production has to be transferred to Y-peaks. The minimum cost flow would then route all of this production to the compound that exhibits the smallest marginal costs

$$\bar{c}_k = \sum_{i \in I_k} p_i d\left(x_i, y_{t(i)}\right). \tag{S8}$$

Here, $t(i) \in I_Y$ denotes the cheapest successor of i that has unreserved sink capacity, and $p_i = v_i/V_X$ is the proportional intensity of peak i. If the production is increased further, the marginal costs remain the same until assignment to one of the corresponding target nodes, say node $t \in I_Y$, hits its capacity, i.e., $\sum_{i\in I_k} f_{i\to t} = w_t$. We now fix this assignment (i.e., reserve the sink capacities occupied so far), and re-evaluate the marginal costs for all compounds. In the next assignment stage, we choose again the cheapest compound and increase source production until the next target node's capacity is exhausted. This procedure is iterated until no more production can be assigned to any compound. Since all flow once assigned will not be changed anymore, displacement of assignment at larger assignment radii as in the example above [*cf.* Fig. S4(a)] do not occur. Nevertheless, over-assignment as for independent compound fitting [*cf.* Fig. S2(b)] is avoided by the simultaneous consideration of all compounds, if all compounds in the mixture are in the library.

S8 In-House Dataset

Individual Compound Library The following 34 individual compounds were used to aquire our in-house dataset: L-methionine (Met), L-glutamine (Gln), L-tryptophane (Trp), L-cysteine (Cys), L-leucine (Leu), L-phenylalanine (Phe), L-isoleucine (Ile), L-tyrosine (Tyr), L-glutamic acid (Glu), L-proline (Pro), nicotinic acid (NA), biotin, D-glucose (Glc), D-mannose (Man), L-rhamnose (Rha), D-xylose (Xyl), raffinose (Raf), octanoic acid (OA), tetradecanoic acid (TDA), malonic acid (MA), succinic acid (SA), pimelic acid (PA), L-tartaric acid (TA), citric acid (CA), 4-phenylbutanoic acid (4-PBA), *trans*-cinnamic acid (tCA), 4-hydroxycinnamic acid (4-HCA), benzoic acid (BA), 4-hydroxybenzoic acid (4-HBA), vanillic acid (VA), anthranilic acid (AA), 1,2,3-benzenetricarboxylic acid (1,2,3-BTCA), 1,2,4-benzenetricarboxylic acid (1,2,4-BTCA) and 9-anthracenecarboxylic acid (9-ACA). For all compounds, stock solutions in dimethyl sulfoxide-d_6 (DMSO-d_6) were prepared at a concentration of 200 mM. Diluted deuterated hydrochloric acid at a final concentration of ∼210 mM was used to increase the solubility of the amino acids. 3 mM working solutions in DMSO-d_6 containing 3-(trimethylsilyl)propionic-2,2,3,3-d_4 acid sodium salt (TSP-d_4, c = 197 µM) as internal standard were prepared for all compounds from these stock solutions.

In addition, we prepared three series (I)–(III), each consisting of three experimental mixtures of the stock solutions:

(I) all 34 compounds (see Sec. S8) were added at identical molar concentrations:

- **(I.a)** 3.0 mM,
- **(I.b)** 0.3 mM,

(I.c) 0.03 mM.

(II) four selected compounds (D-glucose, pimelic acid, 1,2,3-benzenetricarboxylic acid and L-tyrosine) were added at identical molar concentrations:

(II.a) 30.0 mM,

(II.b) 3.0 mM,

(II.c) 0.3 mM.

(III) mixtures of equal volumes of (I.b) and (II.a/b/c), i.e., the four compounds specified for series (II) had higher concentrations in the mixture than the others:

(III.a) (I.b) + (II.a), i.e., 0.15 mM + 15.0 mM,

(III.b) (I.b) + (II.b), i.e., 0.15 mM + 1.5 mM,

(III.c) (I.b) + (II.c), i.e., 0.15 mM + 0.15 mM.

NMR Spectra Aquisiton and Processing ^{1}H,^{13}C HSQC NMR spectra were recorded using the "hsqcetgpsiwt" pulse sequence (Bruker) at 300 K on a Bruker Avance NEO 600 MHz instrument equipped with a 5 mm TCI cryoprobe. Spectra were acquired after 32 dummy scans with different numbers of scans as listed in Tab. S2. For further analysis, we normalized the spectral intensities with respect to the corresponding number of scans.

Table S2: Number of scans for different compound and mixture spectra.

Sample	Number of scans
Individual compounds	4
Mixture (I.a)	8
Mixture (I.b)	16
Mixture (I.c)	64
Mixture (II.a)	4
Mixture (II.b)	8
Mixture (II.c)	16
Mixture (III.a)	8
Mixture (III.b)	64
Mixture (III.c)	32

Time-domain data points were set to 2048 (f2) and 256 (f1). The spectral witdh was set to 9.62 kHZ (f2) and 25.6 kHz (f1). The acquisition times were set to 0.1065 s (f2) and 0.0050 s (f1), respectively, and the relaxation delay was set to 2 s. Spectra were further processed in TopSpin (version 4.1.4, Bruker). Prior to Fourier transform, spectra were zerofilled to

1024 (f1) times 1024 (f2) datapoints. Automatic phase and baseline corrections were used. Spectra were referenced by setting the TSP-d_4 signal to 0.00(f1)/0.00(f2) ppm. Peaks were automatically picked and integrated in the range from 0–10.5 ppm (f2) and 5–165 ppm (f1) and the results manually corrected, if necessary. Spectral grid data was stored in the ASCII file format after opening them with ACD/Spectrus Processor (version 2021.2.2, ACD/Labs).

S9 Supplementary Results: Compound Detection

To measure the distance of two points $x = (x_H, x_C)$ and $y = (y_H, y_C)$ in spectral coordinates for ^{1}H, ^{13}C HSQC spectra, we use the euclidean metric but scale the ^{13}C dimension by the factor 10 to account for its larger chemical shift scale. That is,

$$d(x, y) = \sqrt{(x_H - y_H)^2 + \left(\frac{x_C - y_C}{10}\right)^2}. \tag{S9}$$

A ball $B_r(x) = \{y \mid d(x, y) \leq r\}$ in this metric represents an ellipse in the frequency domain, *cf.* Fig. S7 and Fig. 5(a) of the main text.

In order to rank the performance of the different methods, we count the number of true and false positives (referred to as TP and FP, respectively) and false negatives (FN) associated to the method's prediction. From these numbers, we calculate the F1 score (F_1), which is defined as

$$F_1 = \frac{2 \times \text{recall} \times \text{precision}}{\text{recall} + \text{precision}} \tag{S10}$$

with recall $= TP/(TP + FN)$ and precision $= TP/(TP + FP)$.

Table S3 reports the details of the performance of the different algorithms at the compound detection task, *cf.* main text, Fig. 3.

For setup A (*cf.* Tab. 1 of the main text), Fig. S6 provides more details for the dependence of F1, recall and precision on the detection threshold ϑ and the assignment radius r. Panel (a) shows the results averaged over all mixtures N925, N987, and N988, and panels (b)–(d) show the separate results for the individual mixtures. While all display similar shapes for the F1 score distributions, it is apparent that mix N925 poses a greater difficulty for the algorithm. The maximal F1 score for N925 ($F_1 = 0.71$ at $r = 0.04$ and $\vartheta = 0.002$) is significantly lower than the values obtained for the other mixtures, such that its lower scoring when optimizing the average F1 score over all mixtures (*cf.* Fig. 3 of the main text and S3) is not primarily a consequence of parameter choice.

Let us consider in more detail, where the reconstruction of N925 fails. The classification task (see main text) is affected by two types of errors: compounds that are not contained

Table S3: True positives (TP), false negatives (FN) and false positives (FP) along with the associated precision, recall and F1 scores for the mixtures N925, N987 and N988. Best results for precision, recall and F1 score are highlighted for each mixture. Values for MetaboMiner, COLMAR-HSQC and SMART-Miner are taken from.Kim et al.[8]

Method	TP	FN	FP	Precision	Recall	F1 score
**N925**						
MetaboMiner	15	12	8	0.65	0.56	0.60
COLMAR-HSQC	18	9	3	**0.86**	0.67	**0.75**
SMART-Miner	19	8	8	0.70	**0.70**	0.70
MCF-A	17	10	6	0.73	0.63	0.68
MCF-B	16	11	6	0.72	0.59	0.65
MCF-C	18	9	6	0.75	0.67	0.71
MCF-D	15	12	3	0.83	0.56	0.67
**N987**						
MetaboMiner	9	12	0	**1.00**	0.43	0.60
COLMAR-HSQC	9	12	1	0.90	0.43	0.58
SMART-Miner	14	7	5	0.74	0.67	0.70
MCF-A	18	3	5	0.82	**0.86**	0.84
MCF-B	18	3	4	0.86	**0.86**	**0.86**
MCF-C	18	3	5	0.82	**0.86**	0.84
MCF-D	15	6	2	0.88	0.71	0.79
**N988**						
MetaboMiner	16	8	3	0.84	0.67	0.74
COLMAR-HSQC	10	14	0	**1.00**	0.42	0.59
SMART-Miner	15	9	7	0.68	0.63	0.65
MCF-A	21	3	1	0.95	0.88	0.91
MCF-B	20	4	1	0.95	0.83	0.89
MCF-C	22	2	1	0.96	**0.92**	**0.94**
MCF-D	19	5	0	**1.00**	0.79	0.88

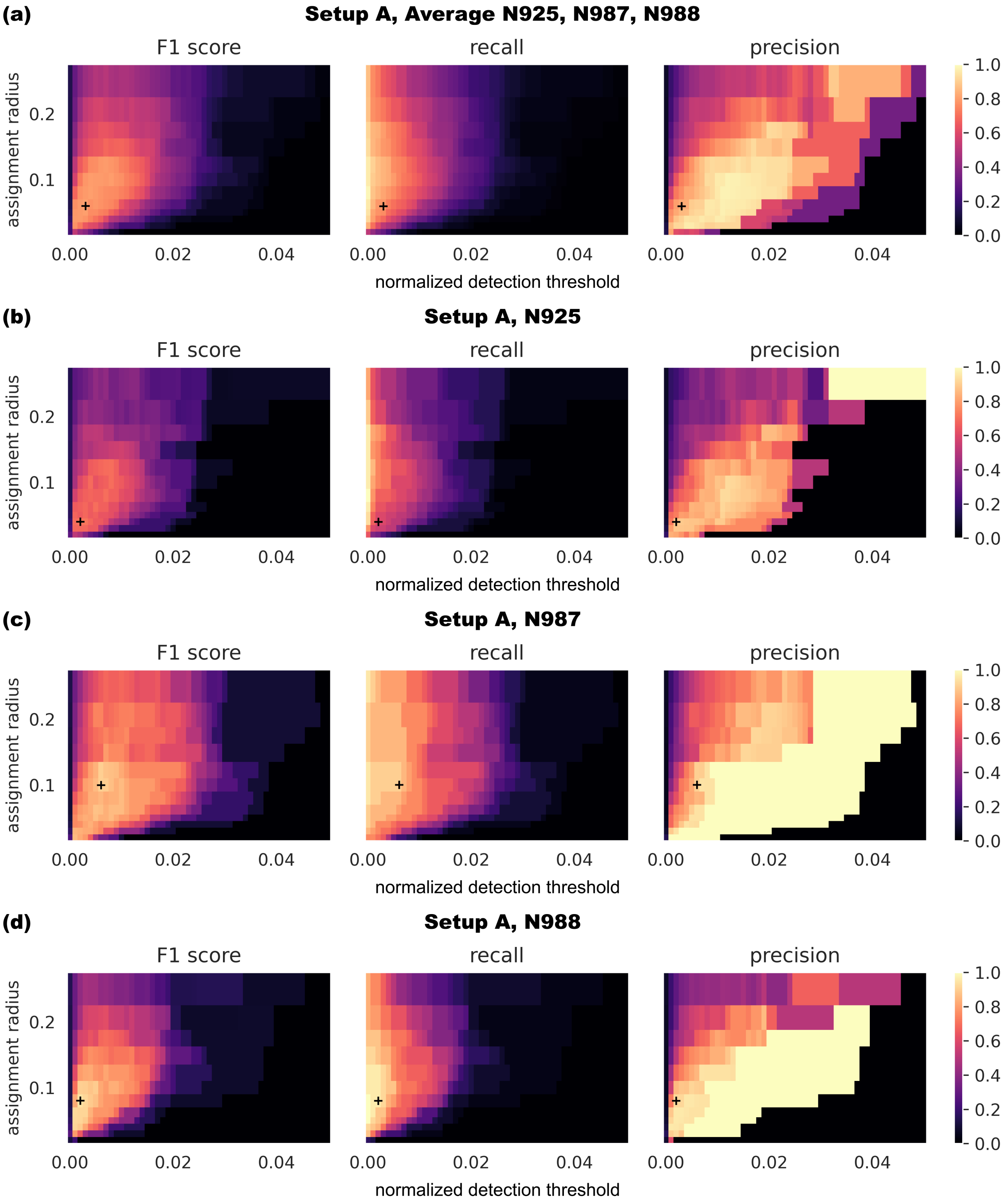

Figure S6: (a) Average and (b)–(d) individual performance of setup A for mixtures N925, N987, and N988.

in the mixture may be falsely detected (false positives), or compounds that are contained remain undetected (false negatives). The mixture N925 contains 27 compounds. Using $r = 0.06$ and $\vartheta = 0.003$ and setup A, the flow method correctly detects 17, falsely rejects 10 compounds, and falsely indicates the containment of 6 compounds from the library of 501 compounds. This gives a recall of 0.63, a precision of 0.73 and $F_1 = 0.68$. In Fig. S7(a)–(d) we show different regions of individual compound spectra projected on the spectrum of mixture N925. Compound peaks are depicted as a blue dots, surrounded by an ellipse reflecting the assignment radius. In (a), as an example for one of 17 successful identifications, we show the spectrum of L-lysine which comprises six peaks in total (only four are in the displayed region). For each compound peak, a mixture peak is present within the assignment ellipse, thus allowing flow to be assigned to the lysine nodes. In the optimal assignment this flow exceeds a critical volume, therefore lysine is classified as being contained in the mixture. Three false positives were observed for N925, one (6-dimethylaminopurine) has only one peak in the scanned area [spectra were recorded from 0 to 140 ppm (^{13}C dimension) and from 0 to 10 ppm (^{1}H dimension), respectively], another (taurine) has two, both matching mixture peaks well. This illustrates that a small number of peaks can coincide by chance, which leads to more false detections of compounds with fewer peaks. The third false positive is sucrose [Panel (b)], which has 11 peaks (7 shown) all matching mixture peaks. Although the data states that sucrose is not contained in N925, a coincidental match seems rather unlikely. Even if the positive indication is false, it is compelling for this case. The same misclassification of sucrose is consequently made by other algorithms, e.g., MetaboMiner. Panel (c) and (d) show examples of false negatives. In (c) an isolated peak of the spectrum of *cis*-aconitic acid is shown, which does not match any peaks of the mixture spectrum. Even if other peaks of the compound would match mixture peaks, the one mismatch would block any assignment. Spectrum peaks for tyrosine, see (d), seem to correspond to some weak signals in the mixture spectrum. Although these weak peaks appear slightly shifted (similar for the other three peaks outside the displayed area), some weight is assigned in this case. However, the total assignment remains below the detection threshold. Thus, the compound is not detected, either because the concentration of tyrosine in N925 is low, or other effects attenuate the peak intensity in the mixture spectrum.

Figure S8(a)–(c) shows the distributions of the average F1 score, recall and precision across mixtures N925, N987 and N988 for setups B-D.

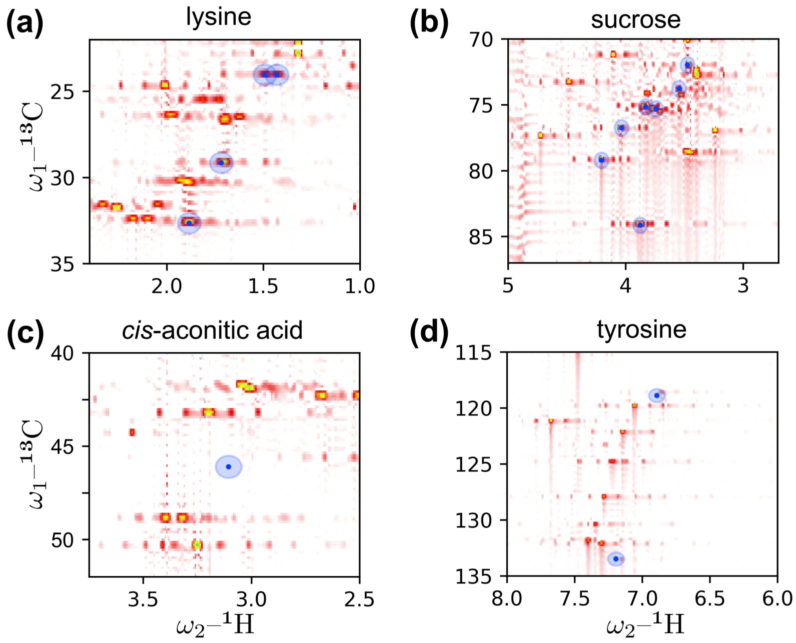

Figure S7: Details for compound identification in mixture N925. The pixel colors encode the signal intensity. Overlay of individual compound peaks (blue dots) with a shaded ellipse indicating the assignment radius of $r = 0.06$: (a) lysine (true positive); (b) sucrose (false positive); (c) and (d) *cis*-aconitic acid and tyrosine (false negatives).

S10 Supplementary Results: Plasma Sample

We tested the performance of our MCF method variants A-D on a plasma sample provided by the Wishart lab[9] and compared the performance with that of MetaboMiner, COLMAR and SMART-Miner (*cf.* Fig. 4 of the main text). Two variations of this sample were used: one recorded at a physiological pH of 7.3 and one recorded at a more basic pH of 8.8, designated as N926 and N907, respectively. To ensure comparability with the MetaboMiner publication[9] the F1 scores (as well as recall and precision, *cf.* Tab. S4) were calculated by treating the detected compounds that have been independently confirmed as true positives, the confirmed compounds that have not been detected as false negatives and the detected compounds that have not been confirmed as false positives. We used two tools from the COLMAR framework[10]: COLMARm to process the grid spectra and COLMAR-HSQC for the peak list from the MetaboMiner data set. We also tested MCF-D on the peak lists obtained from Deep Picker [MCF-D (dp)] during processing the grid spectra with COLMARm in order to evaluate the influence of state-of-the-art peak picking.

For the comparison, we used the optimal values of ϑ and r for each MCF setup. Using the COLMAR web interface[10], we referenced the spectra using alanine, leucine, and lactate for COLMAR-HSQC (using the MetaboMiner peak list). In case of COLMARm, we also

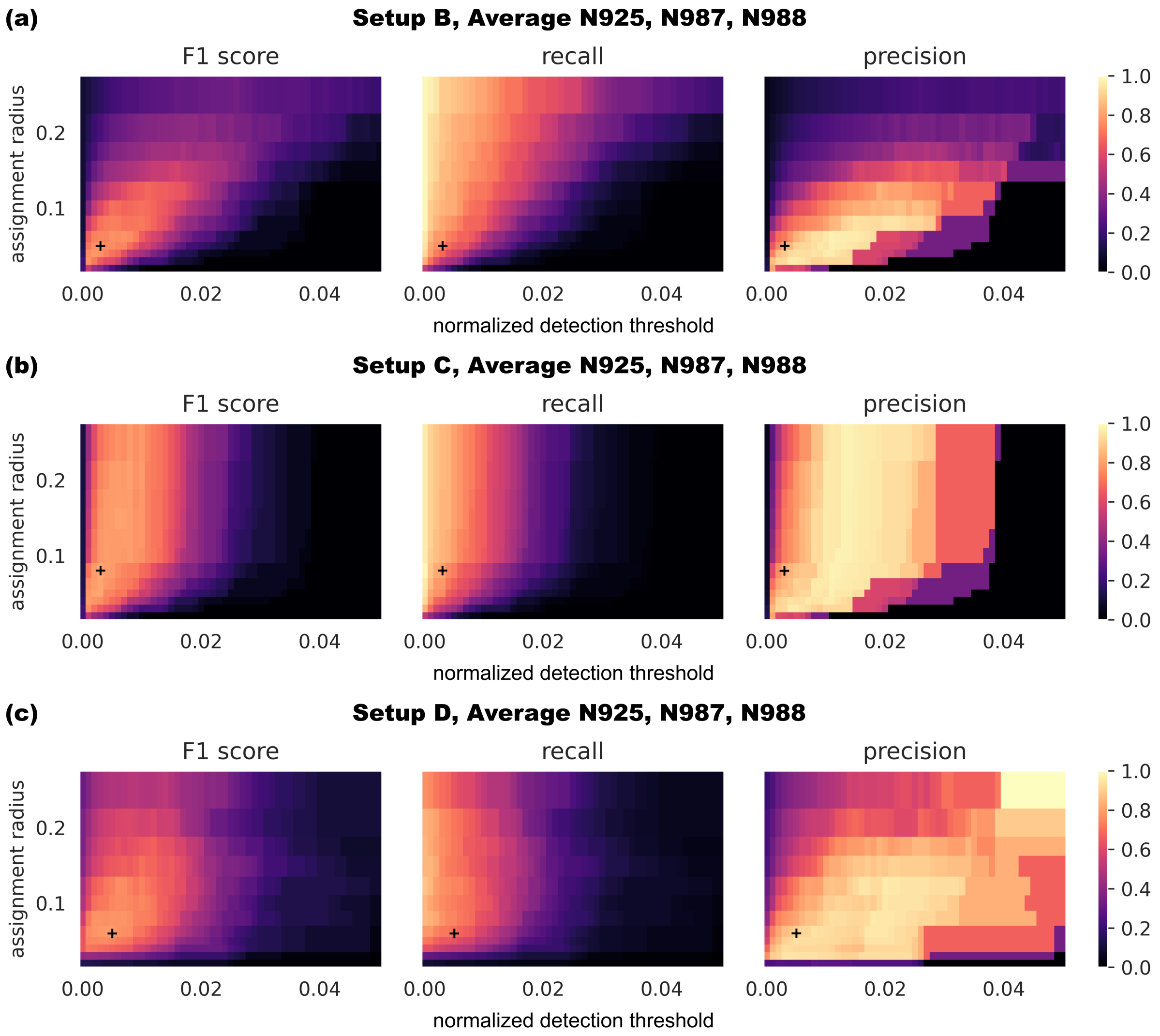

Figure S8: Average perfomance of (a) setup B, (b) setup C, and (c) setup D for mixtures N925, N987, and N988.

included glucose for referencing. Please note that glucose was excluded for referencing in COLMAR-HSQC because one of its peaks was not included in the MetaboMiner peak list, which led to issues when including glucose. When matching the peaks to the database we used default parameters but optimized the "Matching ratio" cutoffs to 0.8 for COLMAR-HSQC and 0.9 for COLMARm to obtain highest F1 scores. For MetaboMiner, we report the performance stats provided by Xia et al.[9] for N926 and ran MetaboMiner on N907 with chemical shift tolerances yielding an optimized F1 value (0.04 ppm and 0.40 ppm for ^{1}H and ^{13}C, respectively) with the full Biofluid library. SMART-Miner does not require parameter choices. Results were obtained using the google Colab Notebook provided by the authors.[11]

It is worth mentioning that the content list provided for the plasma sample contained urea, which does not display any signals in an HSQC spectrum due to missing C–H bonds. However, for comparison with MetaboMiner urea was included as a false negative in the scores for all other methods.

Table S4: True positives (TP), false negatives (FN) and unconfirmed detections (FP) along with the associated precision, recall and F1 scores for a plasma sample spectrum recorded at pH 7.3 (N926) and pH 8.8 (N907). Best results for precision, recall and F1 score are highlighted for each mixture. Data for MetaboMiner on N926 is taken from Xia et al.[9] all other results were recomputed as described above.

**Method**	**TP**	**FN**	**FP**	**Precision**	**Recall**	**F1 score**
**N926**						
MetaboMiner	16	19	7	0.70	0.46	0.55
**COLMARm**	18	17	15	0.55	0.51	0.53
COLMAR-HSQC	18	17	17	0.51	0.51	0.51
SMART-Miner	15	20	45	0.25	0.43	0.32
MCF-A	15	20	6	**0.71**	0.43	0.54
MCF-B	18	17	17	0.51	0.51	0.51
MCF-C	15	20	7	0.68	0.43	0.53
MCF-D	18	17	14	0.56	0.51	0.54
MCF-D (dp)	19	16	13	0.59	**0.54**	**0.57**
**N907**						
MetaboMiner	10	25	4	**0.71**	0.29	0.41
COLMARm	10	25	10	0.50	0.29	0.36
COLMAR-HSQC	11	24	14	0.44	0.31	0.37
SMART-Miner	8	27	24	0.25	0.23	0.23
MCF-A	12	23	18	0.40	0.34	0.37
MCF-B	9	26	8	0.53	0.26	0.35
MCF-C	12	23	15	0.44	0.34	0.39
MCF-D	20	15	26	0.43	**0.57**	**0.49**
MCF-D (dp)	11	24	8	0.58	0.31	0.41

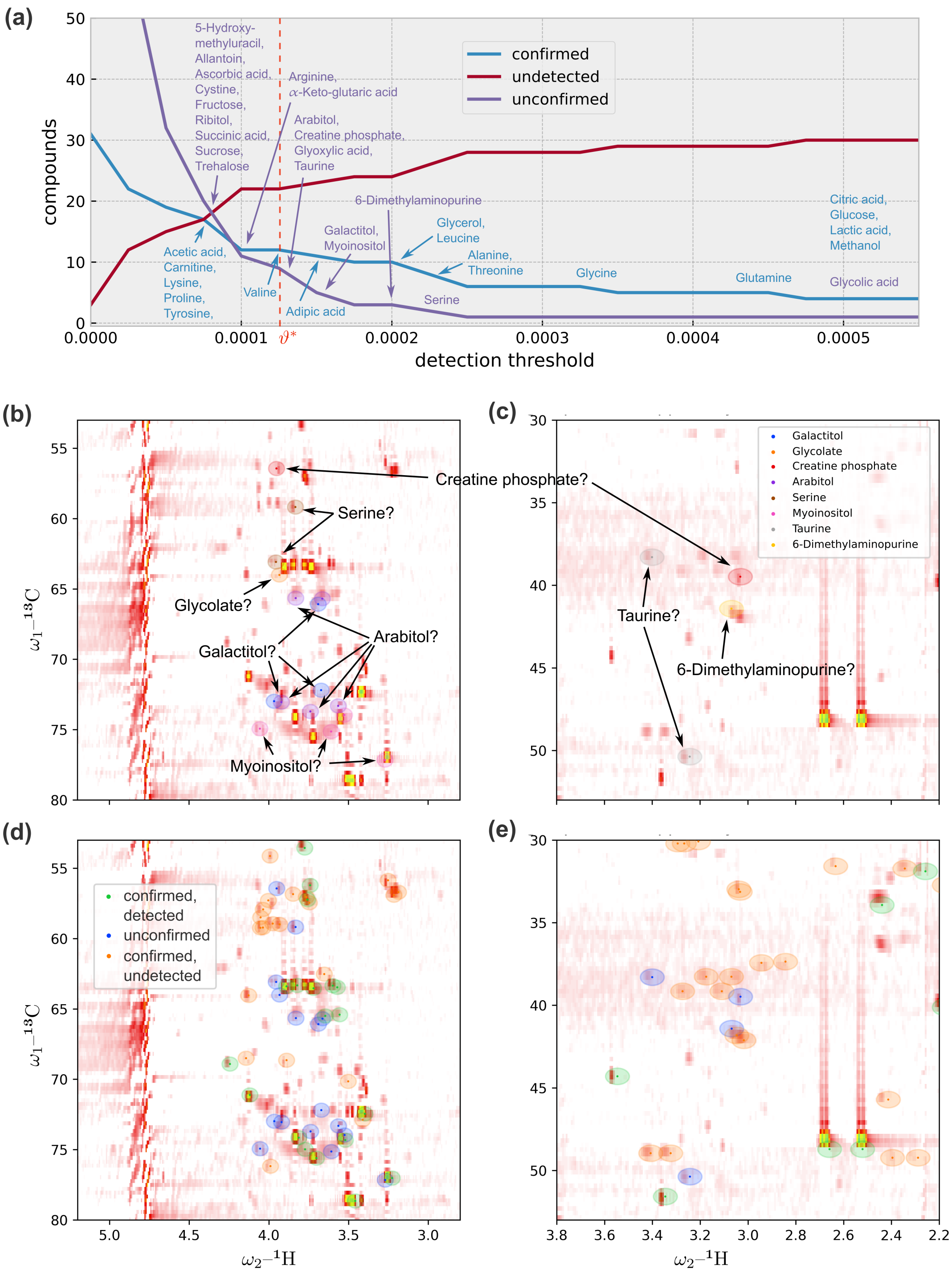

Figure S9: Detected and undetected compounds for method MCF-A on plasma spectrum N926. (a): Detected compounds as a function of the detection threshold for a fixed assignment radius $r = 0.05$ with annotations of confirmed and unconfirmed detected compounds. The red dashed line indicates the value $\vartheta^* = 1.25 \cdot 10^{-4}$ of the detection threshold used for the results shown in (b)–(e). (b) and (c): Spectral areas around peaks of unconfirmed detections for ϑ^*. (d) and (e) same areas with confirmed (detected and undetected) and unconfirmed, but detected, compound peaks for ϑ^*.

Table S5: True positives (TP), false negatives (FN) and unconfirmed detections (FP) along with the associated precision, recall and F1 scores for a plasma sample spectrum recorded at pH 7.3 (N926) and pH 8.8 (N907) using MetaboMiner's "plasma (common)" library[9]. Best results for precision, recall and F1 score are highlighted for each mixture. Data for MetaboMiner is taken from Xia et al.[9] all other results were recomputed as described above.

Method	TP	FN	FP	Precision	Recall	F1 score
**N926**						
MetaboMiner	29	6	6	0.83	0.83	**0.83**
MCF-A	31	4	10	0.76	**0.89**	0.82
MCF-B	31	4	10	0.76	**0.89**	0.82
MCF-C	31	4	10	0.76	**0.89**	0.82
MCF-D	25	10	4	**0.86**	0.71	0.78
MCF-D (dp)	24	11	4	**0.86**	0.69	0.76
**N907**						
MetaboMiner	24	11	4	0.86	0.69	0.76
MCF-A	31	4	10	0.76	0.89	0.82
MCF-B	32	3	11	0.74	0.91	0.82
MCF-C	33	2	11	0.75	**0.94**	**0.84**
MCF-D	22	13	2	**0.92**	0.63	0.75
MCF-D (dp)	20	15	4	0.83	0.57	0.68

For a fixed assignment radius $r = 0.05$, the number of compounds detected by method MCF-A is plotted as a function of the detection threshold in Fig. S9(a). Additional detections occurred when lowering the threshold are annotated for $\vartheta > 0.5 \cdot 10^{-4}$. Panels (b)–(e) show two spectral regions containing peaks of unconfirmed detections (false positives) for a value $\vartheta^* = 1.25 \cdot 10^{-4}$ [*cf.* red dashed line in (a)]. For each compound a sufficiently strong intensity must be present in the target area associated to each of its peaks in order to trigger its detection. Further, the flow optimization may choose to favor one of two compounds "competing" for a common peak. Panels (b) and (c) show peaks of unconfirmed detections (a different color for each compound), while (d) and (e) show all peaks of the confirmed compounds (green if detected, orange if not) and unconfirmed detections (blue). Several of the unconfirmed detections cover multiple peaks (e.g., creatine phosphate, taurine, or serine), which are not explained by the confirmed compounds. For these compounds, the confirmation procedure (whose details are unpublished) should be reviewed to exclude these strong indications for containment. Other cases of unconfirmed detections, where the corresponding compound spectra consist of a single peak within the scanned region of the target spectrum (glycolate and 6-dimethylaminopurine), or whose peaks are located in regions of high peak density (arabitol, galactitol, myoinositol), are more likely a result of coincidence.

S11 Supplementary Results: Quantification

For three experimental series (I)–(III) (*cf.* Sec. S8), we tested the performance for different setups of the MCF-method for quantitative reconstruction of compound concentrations using the same distance (S9) as for compound detection. The results are shown in Fig. S10.

For each experiment, it shows two panels: the dependence of the mean relative error magnitude $\bar{e} = \sum_k |e_k| / N_{\mathrm{mix}}$ (upper panel, N_{mix} being the number of compounds in the mixture) and the percentage of compounds with error e_k within the range $[-0.5, 1.0]$ (lower panel) on the assignment radius r. For all experiments, the performance improves with increasing r within the interval 0.0–0.1 or 0.0–0.2, respectively. Thus, errors decrease and consequently the distribution of errors concentrates within $[-0.5, 1.0]$. For radii beyond the optimal r (i.e., at minimal error), the performance decreases again. This is, however, the part where different setups diverge. For independent compound fits (setup B), the decrease is fastest, as nothing prevents assignment to neighboring peaks. At some point, this leads to an overestimation of concentrations for all compounds. This effect is ameliorated by a simultaneous assignment (setup A), where compounds compete for peaks and thereby restrict extant assignment to individual compounds. However, as the radius increases, displacement errors [*cf.* Fig. S5(d)] increase and impair the overall performance. For all tested cases, the incremental assignment strategy (setup C) proved best to avoid these errors. By fixing assigned flow, it stabilizes the fit and thus the performance for increasing r.

Important experimental parameters for the successful quantification of compounds are their concentrations c_k^* and their number N_{mix}, as well as the number of scans NS used to record the mixture spectra. Concentration and NS impact the signal-to-noise ratio S/N, while N_{mix} increases peak overlaps. Both effects limit the precision of the reconstruction. While the concentration c_k^* is directly proportional to signal strength, the noise intensity scales as $\mathrm{S/N} \sim \mathrm{NS}^{\frac{1}{2}}$. Thus, the compound specific S/N scales as

$$I_k = c_k^* \sqrt{\mathrm{NS}}. \tag{S11}$$

We list these factors and the corresponding minimal relative error magnitudes $\bar{e}$ in Tab. S6. When comparing (I.b) to (I.c) (with $I_k = 1.2$ and $I_k = 0.24$), there is a clear performance drop as $\bar{e}$ nearly doubles for (I.c). For (I.a), although $I_k = 8.4$, there is no distinct improvement over (I.b). Similarly, we see no improvement from (II.b) to (II.a). This suggests, that increasing the number of scans or sample concentration beyond a critical value of I_k, will not lead to any benefit. This critical value seems to depend on N_{mix}, which points to a limitation of performance by the mixture's complexity.

For series (III), the value of $\bar{e}$ is dominated by the errors $|e_k|$ of the 30 compounds added

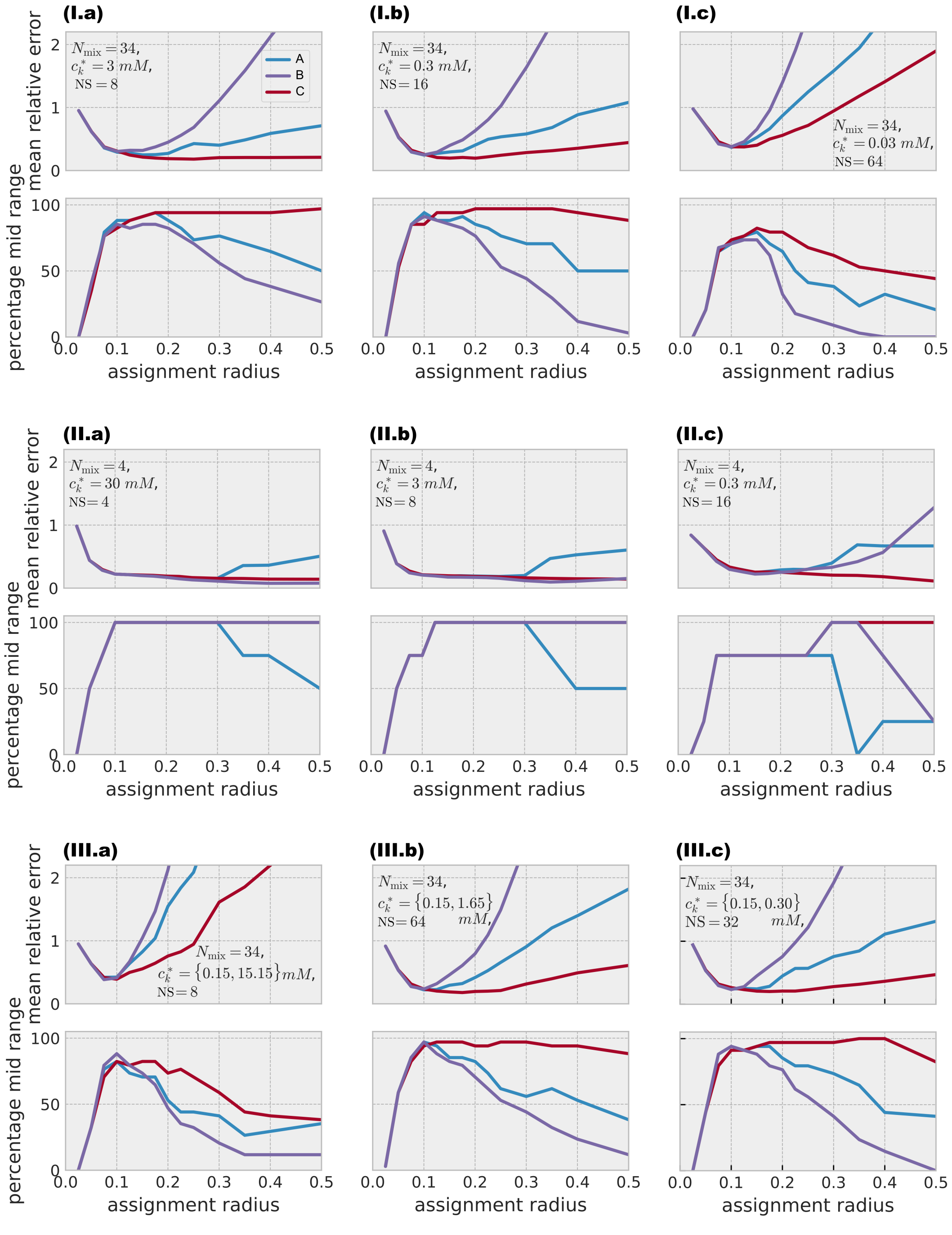

Figure S10: Results for quantitative reconstruction of in-house mixtures. For each experiment and each method setup, the mean of the absolute relative errors $|e_k|$ and percentage of errors in the range $[-0.5, 1.0]$ is shown for a range of assignment radii $r \in [0.025, 0.5]$. The different setups are: simultaneous (MCF-A, blue line), independent (MCF-B, purple line) single pass compound fitting, and simultaneous, incremental assignment (MCF-C, red line).

at concentrations of 0.15 mM. This explains the good correspondence between (III.b) and (I.b). Apart from an insensitivity to values at high I_k, there also seems to be an insensitivity at low I_k. Indeed, the performance is similar for (I.c) and (III.a), whose errors are related to small but different values for I_k (0.24 and 0.42, respectively).

Although not conclusively, these observations suggest a sigmoidal dependence of $\bar{e}$ on I_k. The corresponding parameters (threshold, steepness and asymptotic values) depend on the mixture complexity N_{mix}.

Table S6: Compound specific signal to noise factors, *cf.* Eq. (S11), and mean relative errors $\bar{e} = \text{mean}(|e_k|)$ for different setups. For series III, two values are given for the compound-specific signal-to-noise value I_k, one for the background compounds at concentration 0.15 mM [labeled (bg)], and one for the four spiked compounds with different concentrations [labeled (spiked)], *cf.* Sec. S8.

Experiment	Signal-to-Noise Factors	setup A	setup B	setup C
(I.a)	$I_k = 8.4$	$\bar{e} = 0.25$	$\bar{e} = 0.30$	$\bar{e} = 0.18$
(I.b)	$I_k = 1.2$	$\bar{e} = 0.25$	$\bar{e} = 0.25$	$\bar{e} = 0.20$
(I.c)	$I_k = 0.24$	$\bar{e} = 0.37$	$\bar{e} = 0.38$	$\bar{e} = 0.37$
(II.a)	$I_k = 60$	$\bar{e} = 0.15$	$\bar{e} = 0.08$	$\bar{e} = 0.14$
(II.b)	$I_k = 8.4$	$\bar{e} = 0.18$	$\bar{e} = 0.10$	$\bar{e} = 0.14$
(II.c)	$I_k = 1.2$	$\bar{e} = 0.25$	$\bar{e} = 0.22$	$\bar{e} = 0.11$
(III.a)	$I_k = 0.42$ (bg) and $I_k = 42.9$ (spiked)	$\bar{e} = 0.42$	$\bar{e} = 0.38$	$\bar{e} = 0.39$
(III.b)	$I_k = 1.2$ (bg) and $I_k = 13.2$ (spiked)	$\bar{e} = 0.22$	$\bar{e} = 0.23$	$\bar{e} = 0.18$
(III.c)	$I_k = 0.85$ (bg) and $I_k = 1.7$ (spiked)	$\bar{e} = 0.24$	$\bar{e} = 0.23$	$\bar{e} = 0.20$

We analyzed the linear relationship between predicted and actual concentrations using our in-house dataset and the incremental setup (MCF-C). All compounds demonstrated strong linearity, with R^2 values exceeding 0.95. The only exceptions were 1,2,4-benzenetricarboxylic acid and nicotinic acid, which had R^2 values of 0.61 and 0.94, respectively. Although all compounds exhibited a linear relationship between predicted and actual concentrations, some were consistently underestimated, while a few others were systematically overestimated.

As examples, we show plots for the four compounds used with varying concentrations in the spiking experiments (i.e., D-glucose, L-tyrosine, pimelic acid, benzene-1,3,5-tricarboxylic acid) in Fig. S11(b)—(e) since these compounds cover the widest concentration range analyzed. The predicted concentrations of L-tyrosine, pimelic acid and benzene-1,3,5-tricarboxylic acid did not only linearly correlate with the expected concentrations but also matched the expected concentrations with average errors of 14.3%, 27.5%, and 5.1%, respectively. Conversely, D-glucose exhibited a strong linear relationship ($R^2 = 0.99$), but concentrations were consistently underestimated (with an average error of 42.4%).

Our findings predominantly revealed underestimation rather than overestimation, *cf.*

Fig. S11(a). One important reason for this issue is variations in peak intensities for a given compound, which causes challenges in our current method's implementation. This is because the relative peak intensities in the database spectrum of an individual compound and those in the query spectrum (*cf.* Eq. (6) of the main manuscript) must match. Consequently, a compound's concentration estimate decreases when the intensity of a single peak in the query spectrum is reduced relative to other peaks. For compounds that exist as a single isomer, variations in peak proportions most likely result from differences in the matrix or non-standardized measurement conditions. Additionally, compounds existing as multiple diastereomers may be particularly sensitive to changes in peak proportions. For instance, we observed this effect with the two anomeric forms of D-glucose, whose ratio varied significantly depending on sample concentration and matrix conditions. For these cases, we suggest to deposit individual spectra for all diastereomers in the database.

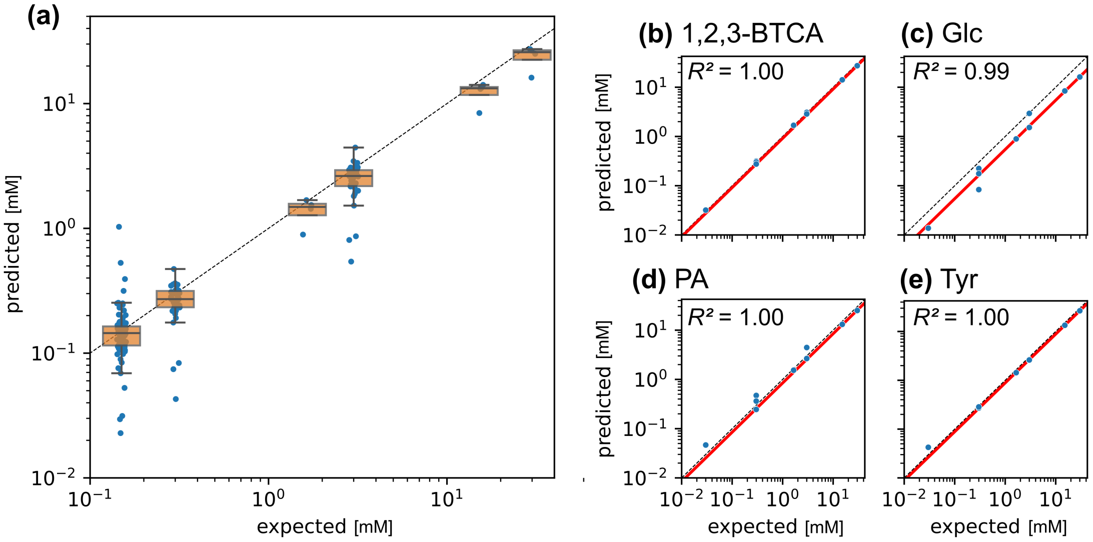

Figure S11: Predicted *vs.* expected concentrations for the incremental fit using setup MCF-C with an assignment radius $r = 0.15$. (a) Data for contained compounds (expected > 0) is shown as scatter plot (with a slight jitter along x-axis for visibility). Boxes show the quartile range Q_1–Q_3 and whiskers extend from $Q_1 - 1.5 \cdot (Q_3 - Q_1)$ to $Q_3 + 1.5 \cdot (Q_3 - Q_1)$, see Waskom[12]. (b)–(e) Uncentered linear regressions for benzene-1,3,5-tricarboxylic acid, D-glucose, pimelic acid, and L-tyrosine. Logarithmic scales were used for better visibility, though the linear regression was performed on the original values.

S12 Computational Requirements

In general, the calculation of an MCF by existing algorithms is of exponential complexity in the worst case and if encountering such a case, finding an optimal solution will be time-consuming. However, the practically observed complexity usually scales much better, see, e.g. Bazaraa et al.[7], Chapter 8. This is the case for our application as well. We have calculated the observed asymptotic complexity for a series of MCF calculations for the grid spectra of the mixtures N925 (at resolution 512x512), N987 (at resolution 512x512), and N988 (at resolution 256x512), with increasing assignment radius r (Fig. S12). For the test, we used a laptop with 16GB RAM (DDR4) and 4 CPUs (i5-8350U CPU@1.70GHz).

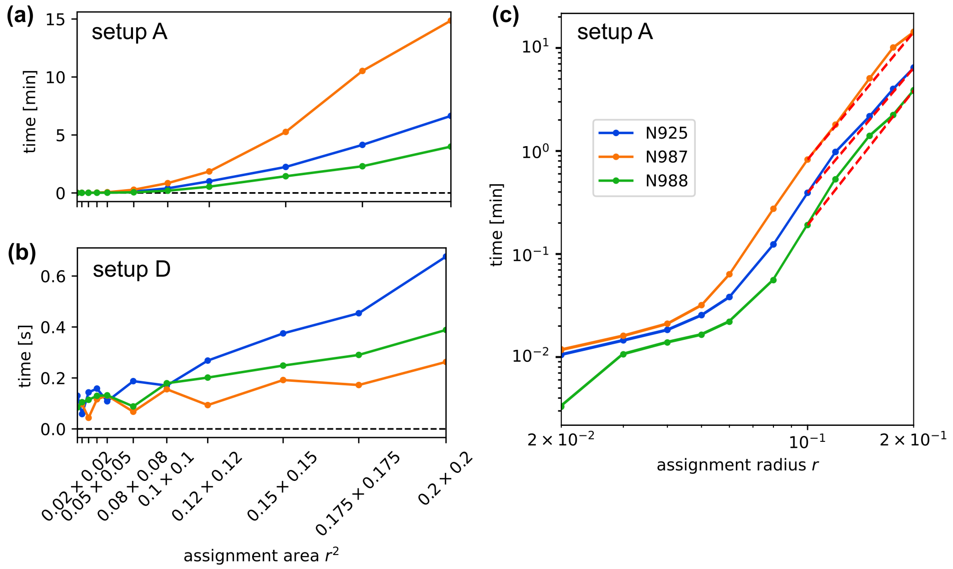

Figure S12: Calculation time (wall time) for the minimum cost flow calculation on a laptop with 4 CPUs for different setups and target spectra of mixtures N925, N987, and N988. (a) Data for setup A (grid target). (b) Data for setup D (peak list target). (c) Same data as (a) but with logarithmic axes.

The number of links in the flow network scales roughly with r^2, such that we are interested in the asymptotic relation between the computation time (wall time) T and r^2. In Figure S12 we show the results of the test runs. In Panels (a) and (b) we show the dependence of the computation time on r^2 for setups A and D, respectively. The square radius scales as the assignment area and thus as the number of links between compound and target peak nodes, which account for the majority of links especially at large values of r. For a grid target spectra, the computation times range from the sub-second scale for very small r over several seconds up to several minutes for a value of $r = 0.2$, see Panel (a). For instance, at the optimal radius $r = 0.08$, we have computation times of 7.5 s (N925), 16.8 s (N987), and 3.4

s (N988). At $r = 0.2$, these times increase to 399 s, 892 s, and 240 s, respectively. For an MCF method operating on peak lists (setup D), the wall time remains consistently below one second [see Panel (b)]. For comparison, we also recorded the computation times of COLMAR and SMART-Miner on the N926 sample. While the web-based compound identification with COLMAR took about 35 s (15 s uploading the ucsf-file, 15 s running DEEP Picker, and 5 s for the database query), SMART-Miner took about 20 min to complete the task on the same laptop specified above. MetaboMiner finds a result after less than one second.

From the timings for the grid target spectra, we find that the computation time scales roughly quadratically with network size (which itself is proportional to r^2). That means $T \sim r^{2\alpha}$ with $\alpha \approx 2$, as we inferred from the log-log slope between $r = 0.1$ and $r = 0.2$ for the test cases [red dashed lines in Panel (c)]. The exact values are $\alpha = 2.0$ (N925), $\alpha = 2.1$ (N987), and $\alpha = 2.2$ (N988).

References

(1) Hitchcock, F. L. The Distribution of a Product from Several Sources to Numerous Localities. *Journal of mathematics and physics* **1941**, *20*, 224–230.

(2) Kantorovitch, L. On the Translocation of Masses. *Management science* **1958**, *5*, 1–4.

(3) Rubner, Y. The Earth Mover's Distance as a Metric for Image Retrieval. *International Journal of Computer Vision* **2000**, *40*, 99–121.

(4) Orlova, D. Y.; Zimmerman, N.; Meehan, S.; Meehan, C.; Waters, J.; Ghosn, E. E. B.; Filatenkov, A.; Kolyagin, G. A.; Gernez, Y.; Tsuda, S.; Moore, W.; Moss, R. B.; Herzenberg, L. A.; Walther, G. Earth Mover's Distance (EMD): A True Metric for Comparing Biomarker Expression Levels in Cell Populations. *PLOS ONE* **2016**, *11*, e0151859.

(5) Hyun, S.; Mishra, A.; Follett, C. L.; Jonsson, B.; Kulk, G.; Forget, G.; Racault, M.-F.; Jackson, T.; Dutkiewicz, S.; Müller, C. L.; Bien, J. Ocean Mover's Distance: Using Optimal Transport for Analysing Oceanographic Data. *Proceedings of the Royal Society A: Mathematical, Physical and Engineering Sciences* **2022**, *478*, 20210875.

(6) Kranstauber, B.; Smolla, M.; Safi, K. Similarity in Spatial Utilization Distributions Measured by the Earth Mover's Distance. *Methods in Ecology and Evolution* **2017**, *8*, 155–160.

(7) Bazaraa, M. S.; Jarvis, J. J.; Sherali, H. D. *Linear Programming and Network Flows*, 1st ed.; Wiley, 2009.

(8) Kim, H. W.; Zhang, C.; Cottrell, G. W.; Gerwick, W. H. SMART-Miner: A Convolutional Neural Network-based Metabolite Identification from ^{1} H- ^{13} C HSQC Spectra. *Magnetic Resonance in Chemistry* **2022**, *60*, 1070–1075.

(9) Xia, J.; Bjorndahl, T. C.; Tang, P.; Wishart, D. S. MetaboMiner – Semi-Automated Identification of Metabolites from 2D NMR Spectra of Complex Biofluids. *BMC Bioinformatics* **2008**, *9*, 507.

(10) Li, D.-W. COLMAR web interface. `https://spin.ccic.osu.edu/index.php/colmar`, accessed on Jul 21, 2024.

(11) Kim, H. SMART-Miner google Colab Notebook. `https://colab.research.google.com/drive/1d_oOzeOkrUXFEYQ4ttBGRTIlzlAnkrn8`, accessed on Jul 21, 2024.

(12) Waskom, M. L. Seaborn: Statistical Data Visualization. *Journal of Open Source Software* **2021**, *6*, 3021.
